# Polytrimethylenimines: Highly Potent Antibacterial Agents with Activity and Toxicity Modulated by the Polymer Molecular Weight

**DOI:** 10.1021/acs.biomac.3c00139

**Published:** 2023-04-24

**Authors:** Julita Pachla, Rafał J. Kopiasz, Gabriela Marek, Waldemar Tomaszewski, Agnieszka Głogowska, Karolina Drężek, Sebastian Kowalczyk, Rafał Podgórski, Beata Butruk-Raszeja, Tomasz Ciach, Jolanta Mierzejewska, Andrzej Plichta, Ewa Augustynowicz-Kopeć, Dominik Jańczewski

**Affiliations:** † Faculty of Chemistry, Warsaw University of Technology, Noakowskiego 3, 00-664 Warsaw, Poland; ‡ Department of Microbiology, National Tuberculosis and Lung Diseases Research Institute, Płocka 26, 01-138 Warsaw, Poland; § Faculty of Chemical and Process Engineering, Warsaw University of Technology, Waryńskiego 1, 00-645 Warsaw, Poland

## Abstract

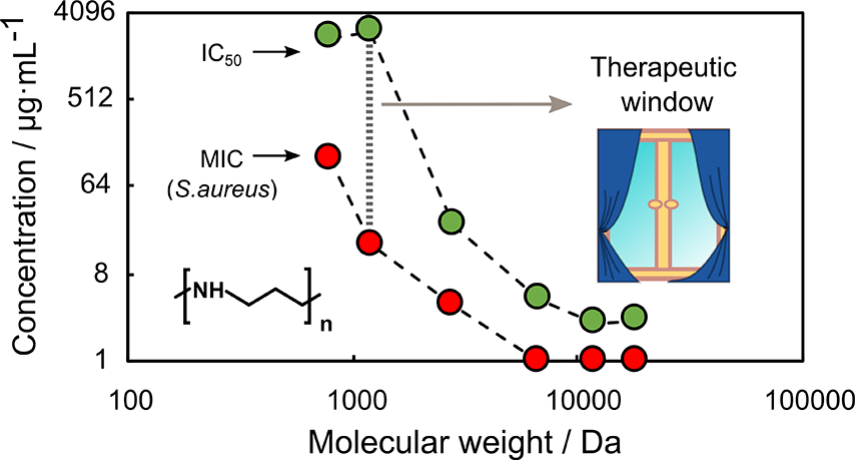

Cationic polymers have been extensively investigated as a potential replacement for traditional antibiotics. Here, we examined the effect of molecular weight (MW) on the antimicrobial, cytotoxic, and hemolytic activity of linear polytrimethylenimine (L-PTMI). The results indicate that the biological activity of the polymer sharply increases as MW increases. Thanks to a different position of the antibacterial activity and toxicity thresholds, tuning the MW of PTMI allows one to achieve a therapeutic window between antimicrobial activity and toxicity concentrations. L-PTMI presents significantly higher antimicrobial activity against model microorganisms than linear polyethylenimine (L-PEI) when polymers with a similar number of repeating units are compared. For the derivatives of L-PTMI and L-PEI, obtained through *N*-monomethylation and partial *N,N*-dimethylation of linear polyamines, the antimicrobial activity and toxicity were both reduced; however, resulting selectivity indices were higher. Selected materials were tested against clinical isolates of pathogens from the ESKAPE group and *Mycobacteria*, revealing good antibacterial properties of L-PTMI against antibiotic-resistant strains of Gram-positive and Gram-negative bacteria but limited antibacterial properties against *Mycobacteria*.

## Introduction

Despite an intense development of antibiotics and other methods of treatment, our densely populated and aging society is exposed to growing threats from microbial diseases. Currently, antibiotic-resistant bacterial infections are responsible for approximately 700,000 deaths annually, and according to predictions, this value will reach 10 million by 2050.^1^ The increasing number of antibiotic-resistant pathogenic strains drives studies toward new antimicrobial agents with a novel mechanism of activity.

One promising area of research is the use of naturally occurring antimicrobial peptides (AMPs)^2^ and their synthetic mimics (SMAMPs).^3−6^ Their mode of action is based on electrostatic attraction to the negatively charged bacterial cell surface and subsequent disruption of its integrity. Such mechanism, based on multiple nonspecific interactions, is potentially less susceptible to development of resistance in comparison to antibiotics in current clinical use.^7,8^

Among many investigated SMAMPs,^3,9,10^ serious research efforts have been devoted to polycations with amine groups allocated within the main polymeric chain, such as polyethylenimine (PEI) and its derivatives.^11,12^ PEI is a close analogue of naturally occurring low-molecular-weight polyamines, like spermine, spermidine, norspermidine, and putrescine. Such short amines are essential compounds in cellular processes of prokaryotic and eukaryotic organisms, acting as, e.g., growth factors and intracellular pH regulators. Importantly, in physiological pH, they are fully protonated, which allows them to bind nucleic acids and other anionic cellular structures via electrostatic interactions. Spermine and spermidine, for example, condense DNA in mammal semen^13^ but also stabilize RNA of viruses. These biogenic polyamines display poor antimicrobial activity,^14^ but they are considered as potent substrates for the synthesis of derivatives with anticancer or antibacterial properties due to the probability of higher biocompatibility compared to fully artificial compounds.^13^ Studies also show that they are able to improve bacterial susceptibility for β-lactam antibiotics.^15^

Derivatives of PEI have been extensively studied as antimicrobial agents,^16,17^ as nonviral transfection vectors,^18,19^ and for other biomedical applications.^19,20^ The results, however, have shown that the polymer exhibits relatively high cytotoxicity, which limits its applications to nonmedical uses such as antibacterial coatings or paints.^20,21^ On the other hand, polytrimethylenimine (PTMI),# an analogue of PEI with a three-methylene spacer between amine groups, has received substantially lower attention. Its dendrimeric form has gained popularity as a nonviral transfection vector and in drug delivery systems,^22,23^ although there are very limited reports on a linear form of PTMI.^24−28^ Different *N*-methylated PTMI derivatives were synthesized;^24,29,30^ however, they have not been the subject of antimicrobial studies. Both PEI and PTMI may be obtained in the branched,^20^ linear,^27,31^ and dendrimeric forms.^23^ Linear PEI (L-PEI) and linear PTMI (L-PTMI) are usually obtained by hydrolysis of poly(alkyl-2-oxazoline)s^32,33^ and poly(alkyl-2-oxazine)s,^28,34^ respectively. Whereas precursors of L-PEI, poly(alkyl-2-oxazoline)s, have already been widely studied for various biomedical applications, poly(alkyl-2-oxazine)s, the precursors of L-PTMI, have been just gathering interest in terms of drug delivery systems^35,36^ and polymer brushes.^37,38^

Polymers have been investigated as antimicrobial agents in various strategies as stand-alone active molecules or conjugates with known active ingredients.^39−41^ The antimicrobial potency and cytotoxicity of polycations depend on many structural parameters, e.g., type of repeating unit (r.u.), type of positively charged group, hydrophilic–lipophilic balance, molecular architecture, and average molecular weight (MW).^42^ The impact of MW on the antimicrobial activity of polymers has been studied for several types of polycations. Reported results indicate that such correlation is highly dependent on the type of polymer and that an increase of MW may lead to an increase,^43−46^ a decrease,^47−50^ or no significant change^51−55^ of antimicrobial activity. Molecular weight also affects the hemolytic and cytotoxic activity of polymers, often leading to the lowered selectivity of materials with a higher polymerization degree.^53,56,57^

The antimicrobial activity of L-PEI has been the subject of several studies;^11,58^ however, the antimicrobial activity of L-PTMI has never been tested. In this work, a series of L-PTMI with different MWs, and L-PEI as a benchmark, were synthesized and characterized. The obtained L-PEI and L-PTMI were additionally subjected to exhaustive *N*-monomethylation and subsequent partial *N,N*-dimethylation. Such library of novel L-PTMI and L-PEI derivatives allowed us to study the influence of MW, type of cationic group, and positive charge density on their antimicrobial activity, including antibiotic resistance species and *Mycobacterium*, and toxicity toward mammalian cells. Presented results reveal the key role of MW in fine-tuning the selectivity of L-PTMI and unprecedentedly high antimicrobial activity of this polycation.

## Experimental Section

### Materials

Reagents were purchased from Sigma-Aldrich, Acros Organics, Alfa Aesar, or Fluorochem. The AR grade solvents were purchased from Chempur or POCH. Deuterated solvents were purchased from Euroisotop. 2-Methyl-2-oxazoline 98% (MeOx, Sigma-Aldrich) and methyl tosylate 98% (MeOTs, Sigma-Aldrich) were additionally purified by distillation over CaH_2_ and stored under an argon atmosphere. Acetonitrile used for polymerization was dried over molecular sieves (13×) and stored under an argon atmosphere. All other reagents were used without further purification. Water was purified using the Milli-Q system (Millipore).

The freeze-drying process was performed using the Labconco FreeZone 2.5 L Benchtop Freeze Dry System. Broths for antimicrobial assays were prepared using the commercially available Mueller–Hinton broth powder (Biocorp), Sabouraud broth (SAB) powder containing 2% dextrose (Merck), and Mueller–Hinton Agar (MHA) powder (Biocorp). Phosphate-buffered saline solution (PBS, pH 7.4) was prepared from the concentrated solution (Fisher BioReagents) by 10 times dilution using Milli-Q water. American Type Culture Collection (ATCC) bacterial and yeast strains were used as model organisms, namely, *Escherichia coli* (ATCC 8739), *Staphylococcus aureus* (ATCC 6538), *Candida albicans* (ATCC 10231), and *Mycobacterium tuberculosis* H37Rv strain (ATCC 25618). Clinical isolates, i.e., *Mycobacterium tuberculosis* Spec. 7153 (isoniazid resistant, INH-R); *Mycobacterium tuberculosis* Spec. 262/19 (rifampicin resistant, RMP-R); *Mycobacterium tuberculosis* Spec. 561/17 (INH-R and RMP-R); *Mycobacterium tuberculosis* Spec. 5419; *Escherichia coli* Spec. 2533/22; *Pseudomonas aeruginosa* Spec. 5037/22; *Acinetobacter baumannii* Spec. 3109/19 (multidrug resistant, MDR); *Klebsiella pneumoniae* Spec. 5223/21; *Klebsiella pneumoniae* Spec. 5223/21 (extended-spectrum β-lactamase, ESBL); *Klebsiella pneumoniae* Spec. 6024/21 (ESBL; ciprofloxacin resistant, CL-R); *Klebsiella pneumoniae* Spec. 3402/21 (New Delhi metallo-β-lactamase, NDM); *Enterococcus faecium* Spec. 6877/20 (vancomycin resistant, VRE); *Staphylococcus aureus* Spec. 2218/21, 3040/21, 2914/21 (methicillin resistant, MRSA); and *Staphylococcus aureus* Spec. 4114/19, 4855/19, 4679/19, 4938/19 (methicillin sensitive, MSSA), were obtained from the collection of pathogenic strains of the Microbiology Department of the National Tuberculosis and Lung Diseases Research Institute (Warsaw, Poland). All of those strains were isolated from patients hospitalized at the National Tuberculosis and Lung Diseases Research Institute between 2017 and 2021 and kept frozen at 80 °C prior to use. The mouse fibroblast cell line L929 (ATCC CCL-1) was obtained from the American Type Culture Collection (ATCC). L929 fibroblasts were maintained in Dulbecco’s modified Eagle medium (DMEM, Thermo Fisher Scientific) supplemented with 10% (v/v) fetal bovine serum (FBS, Thermo Fisher Scientific) and antibiotics (100 U·mL^–1^ penicillin and 100 μg·mL^–1^ streptomycin, Thermo Fisher Scientific).

### Instruments

Microwave-assisted reactions were performed in a single-mode microwave reactor (CEM Discover LabMate) equipped with an infrared (IR) temperature sensor. GC analysis was carried out using an Agilent 7820A GC System equipped with an FID detector. A 30 m × 0.32 mm i.d. × 0.25 μm film thickness HP-5 (Agilent 19091J-413) column was used. Helium (99.999%) with a constant flow rate of 6 mL·min^–1^ was used as carrier gas. The injector and detector temperature was set at 250 °C (injection volume 1 μL). The split flow rate was 360 mL·min^–1^, and the split ratio was 60:1. The column oven was kept at 40 °C for 1.0 min, and then the temperature was increased up to 80 °C at a rate of 35 °C·min^–1^ and subsequently to 250 °C at a rate of 75 °C·min^–1^ and held at this temperature for 3 min. After this time, the column oven temperature was increased to 300 °C at a rate of 75 °C·min^–1^ and held for 1 min. The total running time was 9.08 min. ^1^H NMR spectra were recorded using a Varian 400 MHz spectrometer and D_2_O, CD_3_OD, CD_3_CN, or CDCl_3_ as solvents. ^1^H NMR chemical shifts were referenced to the residual signal of the protonated solvent (δ 7.26 for CDCl_3_, δ 3.31 for CD_3_OD, δ 1.94 CD_3_CN, and δ 4.79 for D_2_O).

SEC analysis was performed using an Agilent 1260 Infinity liquid chromatograph equipped with an RID detector, the PSS NOVEMA Max 5 μm analytical 300 × 8 mm column with a precolumn (PSS GmbH), and a mobile phase containing 54/23/23 (v/v/v%) water/methanol/acetic acid and 0.5 M sodium acetate. All chemicals were HPLC grade. To calibrate the method, a series of poly(2-vinylpyridine) standards (PSS GmbH) in the range of molar masses 620 Da–540 kDa were used. Samples, dissolved in the eluent at 5 mg·mL^–1^ concentrations (injection volume 20 μL), were analyzed at 50 °C with a flow rate of 0.4 mL·min^–1^. Molar masses *M*_w_ and *M*_n_ and dispersity *Đ*_M_ were calculated using the Agilent GPC Addon Rev. B.01.02 software.

### Material Synthesis

^1^H NMR spectra of studied polymers and starting materials are presented in Supporting Information (SI) Figures S1–S12. Representative SEC traces are presented in SI Figure S14.

#### 2-*n*-Propyl-2-oxazine (PrOzi)

The process was adapted from the synthetic procedure of 2-substituted cyclic imino ethers.^59,60^ Butyronitrile (278 g, 4.02 mol) was heated to 100 °C with a catalytic amount of zinc acetate (14.7 g, 80.1 mmol), and 3-aminopropan-1-ol (452 g, 6.02 mol) was added dropwise to the stirred mixture. The reaction solution was refluxed for 72 h at 135 °C. Upon cooling to room temperature, the mixture was diluted using 450 mL of dichloromethane and subsequently extracted with 450 mL of water and 450 mL of brine. The product was purified twice by vacuum distillation and subsequent vacuum distillation over CaH_2_, yielding 232 g of a colorless liquid (46%). ^1^H NMR (400 MHz, CD_3_CN) δ (ppm): 4.07 (t, 2H, *-*C*H*_2_*-*N); 3.22 (t, 2H, -C*H*_2_-O); 2.00 (t, 2H, -CH_2_-C*H_2_-C=*); 1.76 (m, 2H, -CH_2_-C*H_2_*-CH_2_-); 1.50 (m, 2H, -CH_2_-C*H_2_*-CH_3_); 0.88 (t, 3H, -C*H_3_*).

### General Procedure for the Preparation of Poly(2-*n*-propyl-2-oxazine) (PPrOzi)

Poly(2-*n*-propyl-2-oxazine)s were synthesized by a typical procedure for CROP of 2-alkyl-2-oxazines.^60−62^ A representative example is described below, and differences in parameters of individual reactions are summarized in Table 1.

**Table 1:** Reaction Parameters for 2-*n*-Propyl-2-oxazine Polymerizations

polymer	reaction time/min	[*M*]_0_:[*I*]_0_^a^	yield/g
PPrOzi_0.8k	180	5:1	1.41 (72%)
PPrOzi_1.2k	90	12.5:1	3.07 (84%)
PPrOzi_3k	40	100:1	0.96 (48%)
PPrOzi_7k	60	100:1	4.36 (51%)
PPrOzi_12k	90	100:1	1.36 (85%)
PPrOzi_18k	120	100:1	1.78 (81%)

a An initial monomer to initiator ratio.

#### Poly(2-*n*-propyl-2-oxazine)_3k (PPrOzi_3k)

A total of 3.3 mL of PrOzi (3.17 g, 24.9 mmol, 1 equiv) and 2.5 mL of dried acetonitrile were added to a dry, magnetic-stirring-equipped microwave reaction vessel under the flow of inert gas. Subsequently, the mixture was stirred, and 1.0 mL of 0.25 M MeOTs (0.01 equiv) stock solution in dried acetonitrile was added. The reaction vessel was placed in the microwave reactor and heated to 120 °C for *t* = 40 min. After microwave heating, the polymerization mixture was cooled to 50 °C and quenched by the addition of methanol. After evaporation of the solvent on a rotatory evaporator, the residue was dissolved in EtOH and precipitated with Et_2_O. The obtained yellow oil was dried in a vacuum, yielding 0.96 g (48%) of the polymer. ^1^H NMR (400 MHz, CD_3_CN) δ (ppm): 3.26 (4H, -C*H*_2_-N-); 2.26 (2H, -C*H*_2_-C=O); 1.77 (2H, -CH_2_-C*H_2_*-CH_2_-); 1.59 (2H, -CH_2_-C*H_2_*-CH_3_); 0.93 (3H, -CH_2_-C*H*_3_).

### General Procedure for the Preparation of Polytrimethylenimines (L-PTMIs)

Linear polytrimethylenimines were synthesized by adapting procedures of the acidic hydrolysis of poly(2-alkyl-2-oxazines).^63,64^ A representative example is described below, and yields and differences in parameters of individual reactions are summarized in Table 2.

**Table 2:** Reaction Yields for Synthesized L-PTMIs

polymer	yield/g
L-PTMI_0.8k	1.03 (87%)
L-PTMI_1.2k	0.99 (92%)
L-PTMI_3k	0.88 (94%)
L-PTMI_7k^a^^,^^b^	2.49 (85%)
L-PTMI_12k^b^	0.76 (86%)
L-PTMI_18k^b^	0.63 (71%)

a 4.00 g of PPrOzi_7k (31.5 mmol of r.u.) and 20 mL of 6 M HCl were used.

b After removing volatiles *in vacuo*, the residue was dissolved in hot water and alkalinized with 8 M sodium hydroxide. After cooling, precipitated L-PTMI was centrifuged and washed with aqueous NH_3_ (12.5% water solution) until the supernatant conductivity was in the order of magnitude of NH_3_ solution conductivity. Then, the white solid was dispersed in distilled water, frozen, and freeze-dried for 72 h. Subsequently, the polymer was dissolved in 6 M HCl (1.1 equiv). The solution was concentrated and dried *in vacuo*, and the residue was dissolved in distilled water, frozen, and freeze-dried for 72 h.

#### Linear Polytrimethylenimine_0.8k (L-PTMI_0.8k)

PPrOzi_0.8k (1.20 g, 9.45 mmol of r.u.) was dissolved in hydrochloric acid (6 mL, 6 M) in a microwave reaction vessel and heated to 140 °C for 2.5 h. The volatiles were removed *in vacuo*, and the residue was dissolved in distilled water, frozen, and freeze-dried for 72 h, yielding 1.03 g (87%). ^1^H NMR (400 MHz, D_2_O) δ (ppm): 3.14 (4H, -C*H*_2_-N-); 2.09 (2H, -CH_2_-C*H*_2_-CH_2_-).

#### Linear *N*-Methyltrimethylenimine (Me-L-PTMI_7k)

L-PTMI *N*-monomethylation was performed according to the Eschweiler–Clarke procedure adapted to postpolymerization modification.^24^ L-PTMI_7k (1.00 g, 17.6 mmol of r.u.), water (5.6 mL), formic acid (2.8 mL), and 35% formaldehyde (2.8 mL) were added to a 25 mL round-bottom flask, and the mixture was refluxed overnight. Upon cooling to room temperature, NaOH (20% water solution) was added to reach pH = 12, and the product was extracted using chloroform. The organic phase was dried using anhydrous MgSO_4_, and the solvent was removed using the vacuum evaporation process. The obtained pale-yellow residue did not require further purification (yield 1.06 g, 85%). ^1^H NMR (400 MHz, CDCl_3_) δ (ppm): 2.32 (4H, -C*H*_2_-N*-*); 2.20 (3H, -C*H*_3_); 1.63 (2H, -CH_2_-C*H_2_*-CH_2_*-*).

#### Linear Poly(*N*-methyltrimethylenimine-*co*-*N*,*N*–dimethyltrimethyleniminium iodide)

To a solution of Me-L-PTMI_7k (0.35 g, 4.86 mmol of r.u.) in methanol (14 mL) in a two-neck-round bottom flask under inert gas, methyl iodide solution in methanol (14 mL) was dosed, and the mixture was refluxed overnight. Then, the solvent was removed using a rotatory evaporator, and the pale-yellow solid was dried overnight in a vacuum. The degree of quaternization (DQ) was determined using ^1^H NMR spectra. Amounts of MeI, yields, and DQ are presented in Table 3.

**Table 3:** Reaction Parameters, Yields, and Degree of Quaternization for Linear Poly(*N*-methyltrimethylenimine-*co*-*N,N*-dimethyltrimethyleniminium iodides)

	MeI			
polymer	equiv	*V*/μL	yield/g	DQ
MePTMI-*co*-Me_2_PTMI_10%_	0.1	31	0.37 (89%)	13%
MePTMI-*co*-Me_2_PTMI_20%_	0.2	62	0.48 (99%)	23%

#### MePTMI-*co*-Me_2_PTMI_10%_

^1^H NMR (400 MHz, CD_3_OD) δ (ppm): 3.53–3.37 (0.55H, -C*H*_2_-N^+^R_3_); 3.27–3.04 (0.76H, C*H*_3_-N^+^R_3_), 2.67–2.40 (3.45H, -C*H*_2_-NR_2_); 2.39–2.21 (2.66H, C*H*_3_-NR_2;_ R_3_N^+^-CH_2_-C*H*_2_-CH_2_*-*N^+^R_3_)*;* 2.06–1.88 (0.57H, R_3_N^+^-CH_2_-C*H*_2_-CH_2_*-*NR_2_)*;* 1.85–1.61 (1.57H, R_2_N-CH_2_-C*H*_2_-CH_2_*-*NR_2_*).*

#### MePTMI-*co*-Me_2_PTMI_20%_

^1^H NMR (400 MHz, CDCl_3_) δ (ppm): 3.91–3.51 (0.92H, -C*H*_2_-N^+^R_3_); 3.42–3.18 (1.47H C*H*_3_-N^+^R_3_); 2.72–2.06 (5.52H, -C*H*_2_-NR_2_; C*H*_3_-NR_2_; R_3_N^+^-CH_2_-C*H*_2_-CH_2_*-*N^+^R_3_)*;* 2.06–1.77 (0.88H, R_3_N^+^-CH_2_-C*H*_2_-CH_2_*-*NR_2_)*;* 1.75–1.45 (0.98H, R_2_N-CH_2_-C*H*_2_-CH_2_*-*NR_2_*).*

#### Poly(2-methyl-2-oxazoline)_4k (PMeOx_4k)

The polymer was synthesized by a typical procedure for CROP of 2-alkyl-2-oxazolines.^65^ Under an argon atmosphere, 6.3 mL of MeOx (6.33 g, 74.4 mmol) and 9.6 mL of dried acetonitrile were added to a dry microwave reaction vessel equipped with a magnetic stirring bar. The mixture was stirred, and 3 mL of 0.25 M MeOTs stock solution in dry acetonitrile was added. The reaction vessel was placed in a microwave reactor and heated to 120 °C for 4 min. After microwave heating, the polymerization mixture was cooled to 50 °C and quenched by the addition of methanol. After evaporation of the solvent and residual monomer on a rotatory evaporator, the polymer was dried under a vacuum and did not require further purification. Yellow oil was obtained with a yield of 5.11 g (84%). ^1^H NMR (400 MHz, CD_3_CN) δ (ppm): 3.41 (4H, -C*H*_2_-N-); 2.06 (3H, -C*H*_3_).

#### Linear Polyethylenimine (L-PEI_4k)

The procedure was adapted from procedures of acidic hydrolysis of poly-2-alkyl-2-oxazolines***.***^63,64^ PMeOx_4k (2.95 g, 34.7 mmol of r.u.) was dissolved in hydrochloric acid (25 mL, 6 M) in a microwave reaction vessel and heated to 140 °C for 1 h. The volatiles were removed *in vacuo*, and the residue was dissolved in hot water (100 mL) and alkalized with 8 M sodium hydroxide. After cooling, precipitated L-PEI was centrifuged and washed with aqueous NH_3_ (12.5% water solution) until the supernatant conductivity was in the order of magnitude of NH_3_ solution conductivity. Then, the white solid was dispersed in distilled water, frozen, and freeze-dried for 72 h (1.17 g, 78%). ^1^H NMR (400 MHz, D_2_O) δ (ppm): 3.50 (4H, -C*H*_2_-N-).

#### Linear Poly(*N*-methylethylenimine) (Me-L-PEI_4k)

L-PEI *N*-monomethylation was performed according to the Eschweiler–Clarke procedure adapted to postpolymerization modification.^24^ L-PEI_4k (0.91 g, 21.1 mmol of r.u.), water (7.0 mL), formic acid (3.5 mL), and 35% formaldehyde (3.5 mL) were added to a 25 mL round-bottom flask, and the mixture was refluxed overnight. After cooling to room temperature, NaOH (20% water solution) was added to pH = 12, and the solution was extracted with chloroform. The organic phase was dried with anhydrous MgSO_4_, and the solvent was removed in a vacuum. The obtained pale-yellow oil did not require further purification (0.97 g, 82%). ^1^H NMR (400 MHz, CDCl_3_) δ (ppm): 2.47 (4H, -C*H*_2_-N*-*); 2.22 (3H, -C*H*_3_).

#### Linear Poly(*N*-methylethylenimine-*co*-*N*,*N*-dimethylethyleniminium iodide)

To a solution of Me-L-PEI_4k (0.35 g, 6.14 mmol of r.u.) in methanol (14 mL) in a two-neck round-bottom flask under inert gas, methyl iodide solution in methanol (14 mL) was dosed, and the reaction was stirred at room temperature for 48 h. Subsequently, the solvent was removed using a rotatory evaporator, and the pale-yellow oil was dried overnight in a vacuum. The degree of quaternization was determined based on ^1^H NMR spectra. Amounts of MeI, yields, and DQ are presented in Table 4.

**Table 4:** Reaction Parameters, Yields, and Degree of Quaternization for Linear Poly(*N*-methylethylenimine-*co-N,N*-dimethylethyleniminium iodides)

	MeI			
polymer	equiv	*V*/μL	yield/g	DQ
MePEI-*co*-Me_2_PEI_10%_	0.1	38	0.44 (100%)	9%
MePEI-*co*-Me_2_PEI_20%_	0.2	76	0.51 (97%)	17%

#### MePEI-*co*-Me_2_PEI_10%_

^1^H NMR (400 MHz, CDCl_3_) δ (ppm): 3.86 (0.36H, -C*H*_2_-NR_3_^+^); 3.49 (0.58H, C*H*_3_-NR_3_^+^); 2.85 (0.35H, NR_3_^+^-CH_2_-C*H*_2_-NR_2_); 2.47 (3.29H, NR_2_-CH_2_-C*H*_2_-NR_2_); 2.30 (0.58H, C*H*_3_-N(*R*)-CH_2_CH_2_-NR_3_^+^); 2.22 (2.30H, C*H*_3_-N(*R*)-CH_2_CH_2_-NR_2_).

#### MePEI-*co*-Me_2_PEI_20%_

^1^H NMR (400 MHz, D_2_O) δ (ppm): 3.45 (0.64H, -C*H*_2_-NR_3_^+^); 3.12 (0.90H, C*H*_3_-NR_3_^+^); 2.88 (0.43H, NR_3_^+^-CH_2_-C*H*_2_-NR_2_); 2.53 (2.92H, NR_2_-CH_2_-C*H*_2_-NR_2_); 2.25 (0.87H, C*H*_3_-N(*R*)-CH_2_CH_2_-NR_3_^+^); 2.19 (1.65H, C*H*_3_-N(*R*)-CH_2_CH_2_-NR_2_).

### Minimum Inhibitory Concentration (MIC) Determination

The broth microdilution method was applied following CLSI M07-A9 Vol. 32 No. 2 (for bacteria) and CLSI M27-A2 Vol. 22 No. 15 (for yeast) protocols. Single colonies of bacteria or yeast were used to inoculate 5 mL of the Mueller–Hinton broth (MHB) or Sabouraud broth, respectively, and the cultures were grown overnight at 37 °C with shaking (240 rpm). The polymer stock solutions (5120 μg·mL^–1^) prepared in Milli-Q water were diluted with the appropriate broth (MHB or SAB) to a concentration of 1024 μg·mL^–1^ and used to prepare a series of different polymer concentrations in the broth (from 512 to 0.25 μg·mL^–1^; 100 μL each) in 96-well plates by the twofold dilution method. Tested concentrations of oligomeric polyamines were within the range from 4096 to 16 μg·mL^–1^. Subsequently, 100 μL of the microbial suspension (2 × 10^5^ CFU·mL^–1^ for bacteria and 2 × 10^3^ CFU·mL^–1^ for yeast) was added to each well. Uninoculated broth, uninoculated broth with polymer solutions, and inoculated broth without any antimicrobial agent were used as controls. Four replicates were performed for each concentration of polymer and the control. The plates were incubated for 20 h at 37 °C. The optical density at 600 nm (OD_600_) was measured using the Synergy H4 Hybrid Microplate Reader (Biotech, Winooski, VT, USA). The recorded MIC value was the lowest concentration of the polymer at which no microbial growth was observed with the microplate reader.

### MIC against *Mycobacterium*

The assay was performed by the twofold serial microdilution method (in 96-well microtiter plates) using a Middlebrook 7H9 Broth medium (Beckton Dickinson) containing 10% of OADC (Beckton Dickinson). The inoculum was prepared from fresh LJ culture in the Middlebrook 7H9 Broth medium with OADC, adjusted to a no. 0.5 McFarland tube, and diluted 1:20. The stock solution of a tested molecule was prepared in water and diluted in the Middlebrook 7H9 Broth medium with OADC by fourfold the final highest concentration to be tested. Compounds were diluted serially in sterile 96-well microtiter plates using 100 μL of the Middlebrook 7H9 Broth medium with OADC. Concentrations of tested agents ranged from 0.25 to 512 μg·mL^–1^. A growth control containing no antibiotic and a sterile control without inoculation were also prepared on each plate. After incubation at 37 °C for 21 days, the MICs were visually assessed as the lowest concentration showing complete growth inhibition of the reference microbial strains. Isoniazid (INH) and rifampicin (RMP) were used as reference drugs.

### Hemolytic Activity Determination

Fresh blood was obtained from the Regional Center for Blood Donation and Blood Treatment in Warsaw. Samples were centrifuged (700*g*, 10 min), the supernatant plasma was rejected, and erythrocytes were washed with ice-cold phosphate-buffered saline (PBS) three times (by centrifuging, 700*g*, 10 min). After final centrifugation, erythrocytes were diluted 10 times with PBS. Polymer solutions were prepared in PBS buffer. Erythrocyte suspensions (500 μL) were added to polymer solutions (500 μL) at investigated concentrations. Samples were incubated for 1 h at 37 °C and then centrifuged (10 min, 700*g*), and hemoglobin release was measured in supernatants using the spectrophotometric method (absorption at λ = 540 nm). PBS buffer and 0.2% Triton X-100 served as negative and as positive controls, respectively. Experiments were performed at the Faculty of Chemical and Process Engineering WUT with OSH approval for research with human blood.

### Cytotoxicity Assay (XTT)

The L929 mouse fibroblast cell line was cultured in 75 cm^2^ cell culture flasks and kept in an incubator at 37 °C with 5% CO_2_. The culture was monitored under the microscope every 2 days, dissociated, and divided when the cells were near 100% confluent. The cell dissociation protocol was based on a 0.25% trypsin–EDTA solution procedure. The cell concentration was counted on a Thoma cell counting chamber (Marienfeld). Polymer samples were dissolved in DMEM (no supplementation with phenol red), sterilized using 0.22 μm PES membrane, and supplemented with fetal bovine serum (FBS) (10% (v/v)) and antibiotics (1% (v/v)). Final polymer concentrations were reached by dilution with DMEM containing FBS and antibiotics. A sterile solution of 0.1% Triton X-100 in DMEM with FBS was prepared as a positive cytotoxicity control; DMEM with FBS and antibiotics was used as a negative control. The L929 cell line was maintained in 96-well plates for 24 h in 10^5^ cells·mL^–1^ concentration and 100 μL of culture medium per each well. Subsequently, DMEM was replaced by the polymer solutions, and after 24 h of cultivation with polymer solutions, cells were rinsed two times by adding 100 μL of PBS.

For the XTT viability assay, 100 μL of DMEM, without phenol red and FBS, and 50 μL of XTT solution with coupling reagent (The Cell Proliferation Kit II, Roche) were added to each culture well and incubated for 4 h. After the XTT was reduced to formazan pigment by living cells, the assay medium, 100 μL from each well, was transferred to a new 96-well plate, and the absorbance at 475 nm was measured in a plate spectrophotometer. The relative cell viability was defined as the ratio between the absorbance from the sample and the absorbance measured for negative control. Experimental data were fitted to the Hill equation, and IC_50_ (half-maximal inhibitory concentration) values were determined. Uncertainty of IC_50_ was expressed as the standard deviation; for details, see SI.

## Results and Discussion

### Polymer Synthesis and Characterization

**Figure 1:**
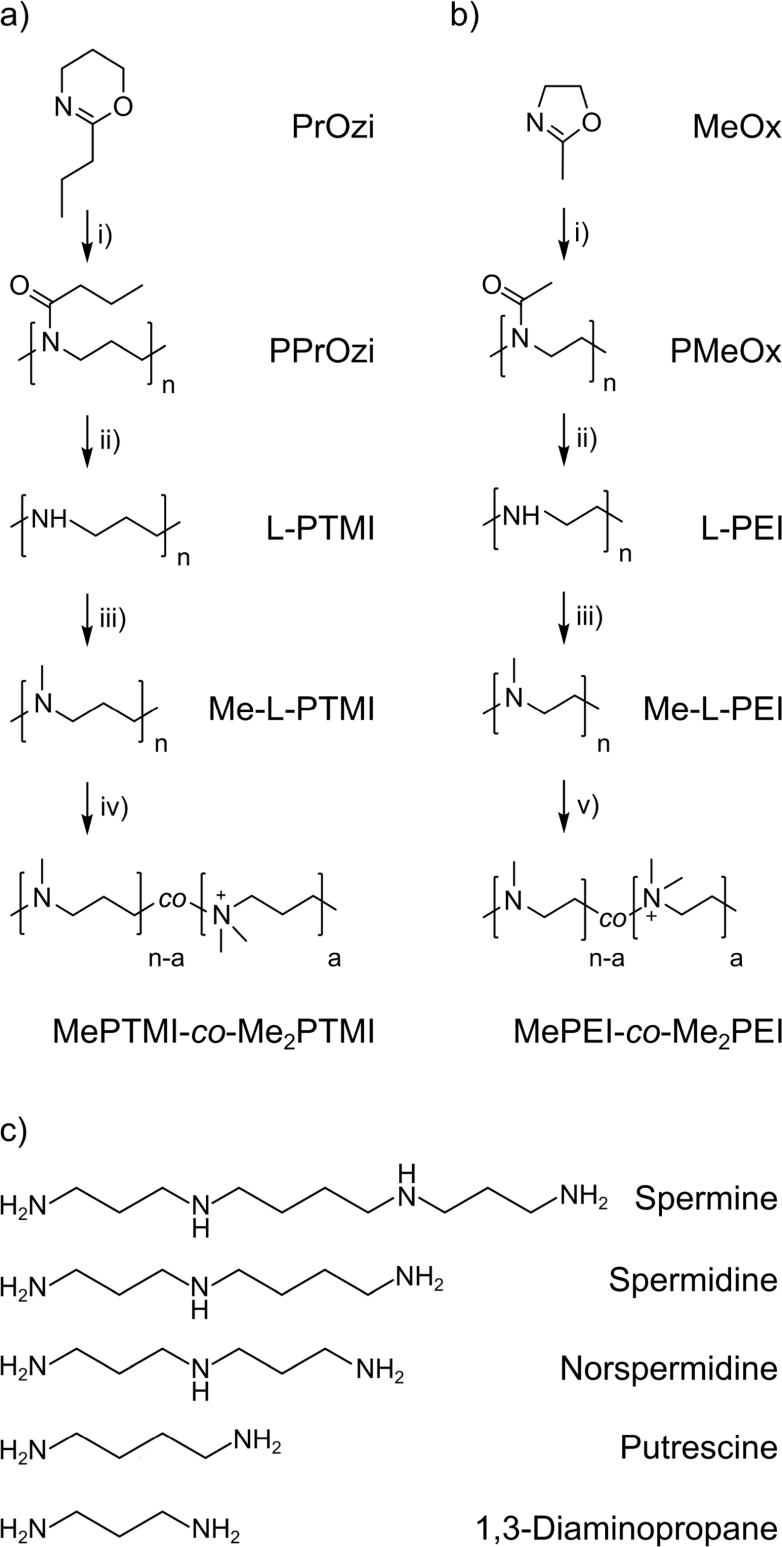
(a) Synthesis pathway of L-PTMI and its derivatives. (b) Synthesis pathway of L-PEI and its derivatives. (c) Oligomeric polyamines used in current studies (reaction parameters: (i) MeOTs, ACN, 120 °C, microwave-assisted reaction; (ii) 6 M HCl, 140 °C, microwave-assisted reaction; (iii) HCOOH, CH_2_O, H_2_O, 90 °C; (iv) MeI, MeOH, reflux; (v) MeI, MeOH, RT (counterions were omitted for clarity).

The main aim of this work was to investigate the antimicrobial activity and toxicity of L-PTMI characterized by different molecular weights and compare them with the biological activity of extensively studied L-PEI. Second, we studied how antimicrobial properties are influenced by postpolymerization modifications of these linear polyamines using tertiary amines and quaternary ammonium salt derivatives. Series of L-PTMIs, with different MWs, were synthesized using a cationic ring-opening polymerization protocol^27,60,66^ of *n*-propyl-2-oxazine and subsequent acidic hydrolysis of obtained poly(*n*-propyl-2-oxazine) (PPrOzi) (Figure 1a). L-PEI was synthesized in a similar process, starting from methyl-2-oxazoline, and used as a reference material.^64^

As reported in the literature, polymerization rates of *n*-alkyl-2-oxazines are approximately 4 times lower than *n*-alkyl-2-oxazolines.^60,67^ Slower propagation leads to a higher contribution of side reactions, such as termination. As a result, the polymerization of oxazines is more challenging to control and more sensitive to traces of impurities. To optimize PTMI polymerization parameters and to obtain PPrOzi with well-defined molecular weights, monomer conversion over the reaction time was monitored with the use of GC. Results indicate that polymerization follows first-order kinetics with respect to the monomer (Figure S13), allowing one to determine the propagation rate (*k_p_* = (5.3 ± 0.1) × 10^3^ L·mol^–1^·s^–1^). In case of shorter PPrOzi, polymerization reactions were performed with complete conversion of the monomer. For larger MWs of PPrOzi, polymerizations were performed in a monomer conversion regime no higher than 70–80% to avoid side reactions and to achieve lower dispersity. MeOx polymerization also follows first-order kinetics (Figure S13, *k_p_* = (41.2 ± 0.8) × 10^3^ L·mol^–1^·s^–1^). Molecular weights (*M*_n_ and *M*_w_) and dispersity (*Đ*_M_) of obtained materials, determined by SEC analysis, are presented in Table 5. Degrees of polymerization (DPs) based on ^1^H NMR and conversion of the monomer via GC are presented in Table S1.

**Table 5:** The Characterization of Obtained Polymers by Size Exclusion Chromatography (SEC)

polymer	*M*_n_/kDa	*M*_w_/kDa	*Đ*_M_
L-PTMI_0.8k	0.80	0.91	1.14
L-PTMI_1.2k	1.2	1.4	1.22
L-PTMI_3k	2.8	3.7	1.33
L-PTMI_7k	6.7	9.6	1.44
L-PTMI_12k	12.1	18.2	1.51
L-PTMI_18k	18.0	27.1	1.51
L-PEI_4k	4.0	5.3	1.30
Me-L-PTMI_7k	3.6	5.4	1.53
MePTMI-*co-*Me_2_PTMI_10%_	4.2	6.5	1.54
MePTMI-*co-*Me_2_PTMI_20%_	3.7	5.5	1.49
Me-L-PEI_4k	3.9	4.8	1.24
MePEI-*co-*Me_2_PEI_10%_	3.6	4.6	1.27
MePEI-*co-*Me_2_PEI_20%_	3.6	4.7	1.31

L-PTMI_7k, containing 116 repeating units (r.u.) by average, and L-PEI_4k (91 r.u.) were used for further polymer modifications, namely, *N*-monomethylation and subsequent partial quaternization of amine groups (Figure 1a,b). The *N*-monomethylation reaction was performed using the Eschweiler–Clarke reaction,^33,68^ leading to fully *N*-methylated polymers (Me-L-PTMI_7k and Me-L-PEI_4k). The last stage of the postpolymerization modification was the partial transformation of *N*-methylamines into *N*,*N*-dimethylammonium groups with the use of MeI to reach the designed degree of quaternization (DQ). All polymers studied in this work had a linear structure. In the case of partially quaternized polymers, the letter “L” was omitted from the acronyms for simplicity. The arbitrarily chosen DQ levels for both polymers were motivated by the limited stability of PEI upon full quaternization. When more than 50% of r.u. of PEI is quaternized, the polymer undergoes degradation due to the proximity of adjacent charged groups within the polymeric chain.^69,70^

### Molecular Weight Influence on Biological Activity

**Figure 2:**
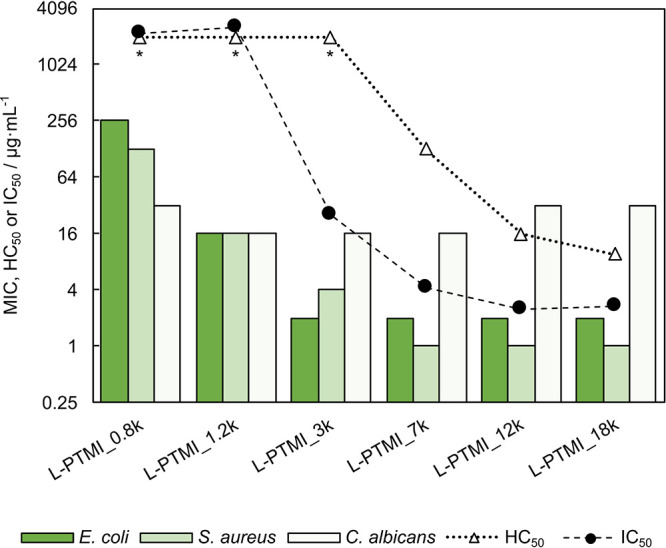
Values of MIC toward model microorganisms, HC_50_ toward erythrocytes, and IC_50_ toward mouse fibroblasts (dotted and dashed lines were drawn only to guide the eye) for L-PTMI with different molecular weights (graphical representation); *HC_50_ > 2000 μg·mL^–1^.

Table 6 summarizes the basic biological activity of investigated molecules. Minimum inhibitory concentrations (MICs) were determined by the broth microdilution method against model microorganisms *E. coli* (Gram-negative bacteria), *S. aureus* (Gram-positive bacteria), and *C. albicans* (yeast). Additionally, toxicity indicators, namely, concentrations causing hemolysis of 50% of red blood cells in the sample (HC_50_) and concentrations inhibiting the metabolism activity of mammalian cells (mouse fibroblasts) by 50% (IC_50_), were assayed. The antibacterial activities of L-PTMI series polymers (*M*_n_ = 0.8, 1.2, 2.8, 6.7, 12.1, and 18.0 kDa) were compared with toxicity data (Figure 2). For bacteria (*E. coli*, *S. aureus*) as well as for both investigated toxicity parameters (IC_50_ and HC_50_), the increase of polymer weight clearly translates into the increased biological activity. Importantly, this trend is characterized by the presence of the threshold value; crossing a certain polymer mass substantially increases the polymer toxicity. The shortest studied L-PTMIs are nontoxic and nonhemolytic materials (up to concentrations of 2 mg·mL^–1^). Above a certain MW, the activity increases significantly, manifested in lower MIC, HC_50_, and IC_50_ values (e.g., MIC 1 μg·mL^–1^ for *S. aureus*). It is noteworthy that the threshold seems to be located at lower MW for MIC values compared to HC_50_ and IC_50_ values. Such difference can potentially be exploited as a source of selectivity for polymeric antimicrobial agents. Selectivity indices have been calculated for all tested materials as the ratio of IC_50_ to MIC against model microorganisms (Table 6). For L-PTMI, selectivity clearly changes with MW and displays a maximum for L-PTMI_1.2 k for all tested model organisms (Figure S17). This trend provides the possibility of tuning antimicrobial and cytotoxic properties by adjusting MW.

**Table 6:** Biological Activity of Investigated Polymers

	MIC^a^/μg·mL^–1^					selectivity index^d^		
polymer	*E. coli*	*S. aureus*	*C. albicans*	HC_50_^b^/μg·mL^–1^	IC_50_^c^*/*μg·mL^–1^	*E. coli*	*S. aureus*	*C. albicans*
L-PTMI_0.8k	256	128	32	>2000^e^ (2%)	2220	8.7	17	69
L-PTMI_1.2k	16	16	16	>2000^e^ (4%)	2570	161	161	161
L-PTMI_3k	2	4	16	>2000^e^ (3%)	25.2	13	6.3	1.6
L-PTMI_7k	2	1	16	125^e^	4.20	2.1	4.2	0.26
L-PTMI_12k	2	1	32	15.6^e^	2.48	1.2	2.5	0.08
L-PTMI_18k	2	1	32	9.40^e^	2.71	1.4	2.7	0.08
L-PEI_4k	16 (31)^11^	16 (8–16)^11,58^	32	>2000^e^ (39%)	660	41	41	21
Me-L-PTMI_7k	16	16	16	>2000 (2%)	240	15	15	15
MePTMI-*co*-Me_2_PTMI_10%_	16	16	16	>2000 (3%)	420	26	26	26
MePTMI-*co*-Me_2_PTMI_20%_	16	16	16	>2000 (3%)	930	58	58	58
Me-L-PEI_4k	32	128	64	>2000 (2%)	600	19	4.7	9.5
MePEI-*co*-Me_2_PEI_10%_	32	64	32	>2000 (3%)	1370	43	21	43
MePEI-*co*-Me_2_PEI_20%_	16	64	16	>2000 (2%)	2000	125	31	125

a Literature values are given in parentheses.

b Hemolytic yield for the polymer concentration of 2 mg·mL^–1^ is given in parentheses.

c Uncertainties of IC_50_ values, expressed as a standard deviation, are summarized in SI (Figure S16 and Table S3).

d Selectivity index = IC_50_/MIC.

e Hemolysis assay was carried out for the polymer solution in 130 mM NaCl instead of PBS due to solubility issues.

The impact of the molecular weight of polycations on their antimicrobial activity was reported previously in numerous papers; the effect is however strongly dependent on the polymeric structure.^44,45,48,52−55^ The cytotoxicity of cationic polymers, similarly to its antibacterial activity, is a function of cationic charge density^56^ and hence typically increases for longer polymers. For example, Tyagi and Mishra^57^ reported that derivatives of polymethacrylamide, bearing a primary amine group in side chains, were highly cytotoxic to human cells for MWs higher than 5 kDa. In few reports, a U-shaped correlation between MW and biological activity is described for cationic amphiphilic polymers.^71−73^ Short oligomers typically display limited antimicrobial and toxic activities, which increase along with MW. It can be explained by a higher overall cationic charge of the macromolecule and multivalent interactions of a single polymeric chain with the cell envelope.^72,74^ For very high molecular weights, activity may be lowered, which is explained by the lower ability of large polymers to penetrate across the cell wall and membrane.^71,75,76^ However, this may also be the effect of lower polymer solubility or aggregation, preventing interactions with the lipid bilayer. Ikeda et al.^43,77^ reported that the activity of polymers bearing biguanide moieties in the main chain against *S. aureus* increases with increasing MWs up to 50 kDa and drops for weights above 120 kDa as a result of limited diffusion across the cell wall. Some reports indicate that the permeability of the cell wall of Gram-positive bacteria is not a concern for molecular weights up to 50–70 kDa.^51,73,78^ In this context, our results for L-PTMI remain in good agreement with published data.

**Figure 3:**
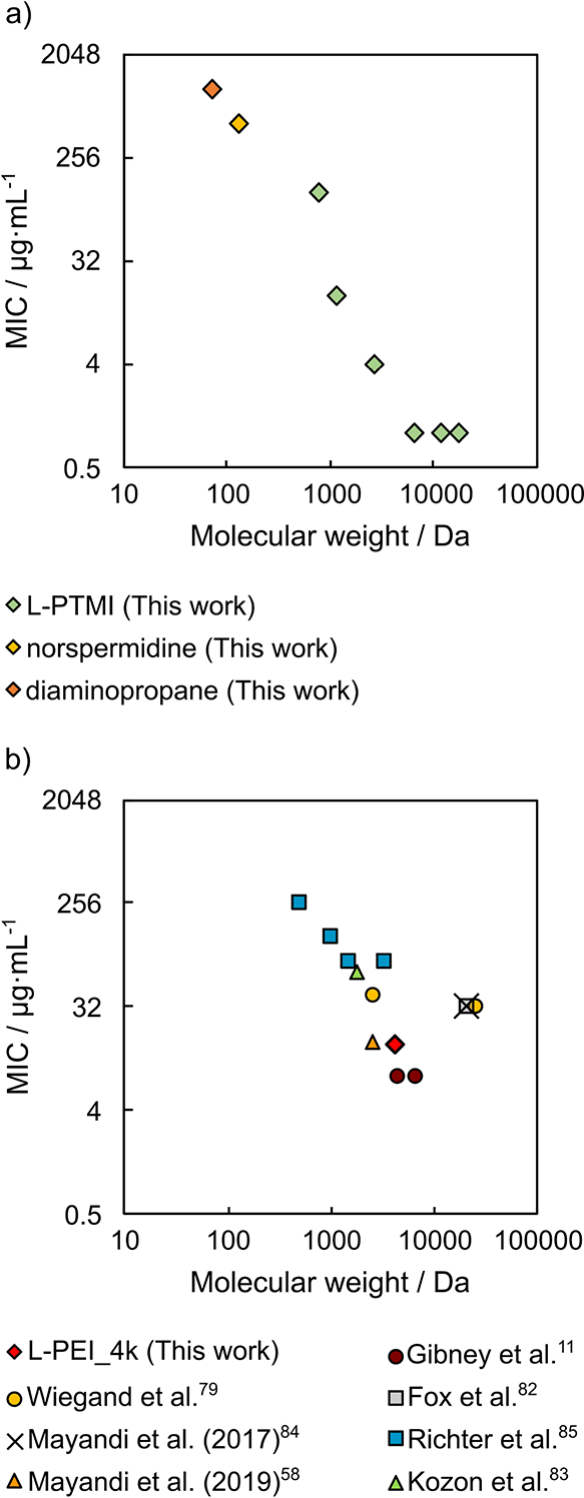
Values of MIC against *S. aureus* as a function of molecular weight for linear polyamines (a) L-PTMI and its oligomeric analogues with a three-methylene spacer (b) L-PEI (majority literature data).

The L-PTMI results were compared against naturally occurring, low-molecular-weight polyamines, namely, spermine, spermidine, putrescine, norspermidine, and 1,3-diaminopropane (Figure 1c), as similar structural analogues.^11,58,79^ All of them display lower antimicrobial activity than L-PTMI, which is particularly well visible for direct structural L-PTMI analogues (norspermidine and 1,3-diaminopropane) (Table 7, Figure 3a, Figure S15) against all tested microorganisms (ca. 4–8 times lower against *E. coli* and *S. aureus* compared to the least active synthesized L-PTMI and 16–64 times lower against *C. albicans*). This well supports the hypothesis that to obtain a bacteriostatic material, a certain minimum number of repeating units are required.^44,80,81^

**Table 7:** Values of MIC for Oligomeric Polyamines^a^

	MIC/μg·mL^–1^		
compound	*E. coli*	*S. aureus*	*C. albicans*
spermine	1024	256	128
spermidine	2048	512	512
norspermidine	2048	512	512
putrescine	2048	1024	1024
1,3-diaminopropane	1024	1024	1024

a MIC literature data for comparison are provided in SI (Table S2).

Additionally, it is worth to highlight that for L-PTMI, MW/activity dependence remains in two regimes (Figure 3a, Figure S15). MIC values decrease linearly in a log–log scale up to a certain polymer mass, and above this mass, MIC values remain constant at the low level. The linear negative correlation may indicate the presence of a single dominating biochemical mechanism responsible for the MW/activity effect in this area.

Even though L-PEI has been relatively widely explored in comparison to L-PTMI, no systematic studies on its antimicrobial activity as a function of molecular weight have been summarized. In several reports, MIC values against model bacterial strains have been determined; however, most of the publications discuss no more than two different polymeric masses (Figure 3b, Figure S15).^11,58,79,82−84^ Richter et al.^85^ tested the activity of four L-PEIs that differed in the number of r.u. and observed a trend of monotonically decreasing MIC values against *E. coli* and *S. aureus* with an increasing number of r.u.. Falco et al.^86^ investigated the transfection efficacy of L-PEI; therefore, they fractionated a highly dispersed polymer and examined its toxicity dependence on molecular weight. The results showed that up to 4 kDa L-PEI seems to be of low toxicity, whereas above 20 kDa, the toxicity is significantly higher.

In our studies, L-PEI_4k (91 r.u.) displays 8- and 16-times lower inhibiting activity against *E. coli* and *S. aureus*, respectively, in comparison to L-PTMI_7k (116 r.u.) (Table 6). Toxicity toward mouse fibroblasts as well as the hemolytic activity of L-PEI_4k was also lower; e.g., IC_50_ is 2 orders of magnitude higher than the value for L-PTMI_7k. There is no simple explanation for such effect, but at least two factors may be considered. The distance between cationic groups, interacting with phospholipids, was hypothesized to influence the inhibitory effectiveness of the molecule as reported Gilbert and Moore^87^ for biguanide polymers. Therefore, one of the possible explanations for the increased activity of L-PTMI is that the presence of an additional methylene group in the alkyl spacer leads to the optimization of the steric arrangement of amine groups in contact with the lipid layer.^11,49,53^ The second possibility could be that the longer alkyl linker between the amine groups enhances the hydrophobic character of the polymer, providing amphiphilic properties to the material. This factor is reported for many SMAMPs to increase both the microbiological activity^88,89^ and red blood cells lysis.^53,55,90^

The influence of the polymer molecular mass on the activity of L-PTMI against *C. albicans* appears to be significantly different than in the case of model bacteria. All of the studied L-PTMIs showed similar moderate activity against *C. albicans* in the tested range of *M*_n_ = 0.8–18 kDa (MIC: 16–32 μg·mL^–1^). This outcome may be puzzling in contrast to MIC values obtained for the same materials against *E. coli* and *S. aureus* (MIC: 1–256 μg·mL^–1^). The different activity of L-PTMI against *C. albicans* is likely related to differences in the structure of microbial cell walls and membranes. Compared to bacteria, the fungal cell wall is thicker and contains chitin, hindering polymer migration to the lipid membrane surface.^91^ The zeta potential of the fungal cell surface is also less negative,^92,93^ further lowering susceptibility to polycationic agents. It is not trivial to explain the lack of a molecular weight effect, but it may be hypothesized that our polymers display antifungal intracellular activity. Some cationic polymers^94,95^ have been reported to enter fungal cells without disrupting the integrity of the fungal membrane.

Unlike in the case of *E. coli* and *S. aureus*, a direct comparison of the antifungal activity between L-PTMI_7k and L-PEI_4k shows a very low difference in MIC values (16 and 32 μg·mL^–1^, respectively). It appears that changes in the length of the alkyl spacer between amine groups, from two to three carbons, do not significantly contribute to increased antifungal activity.

### Biological Activity of L-PTMI and L-PEI Derivatives

**Figure 4:**
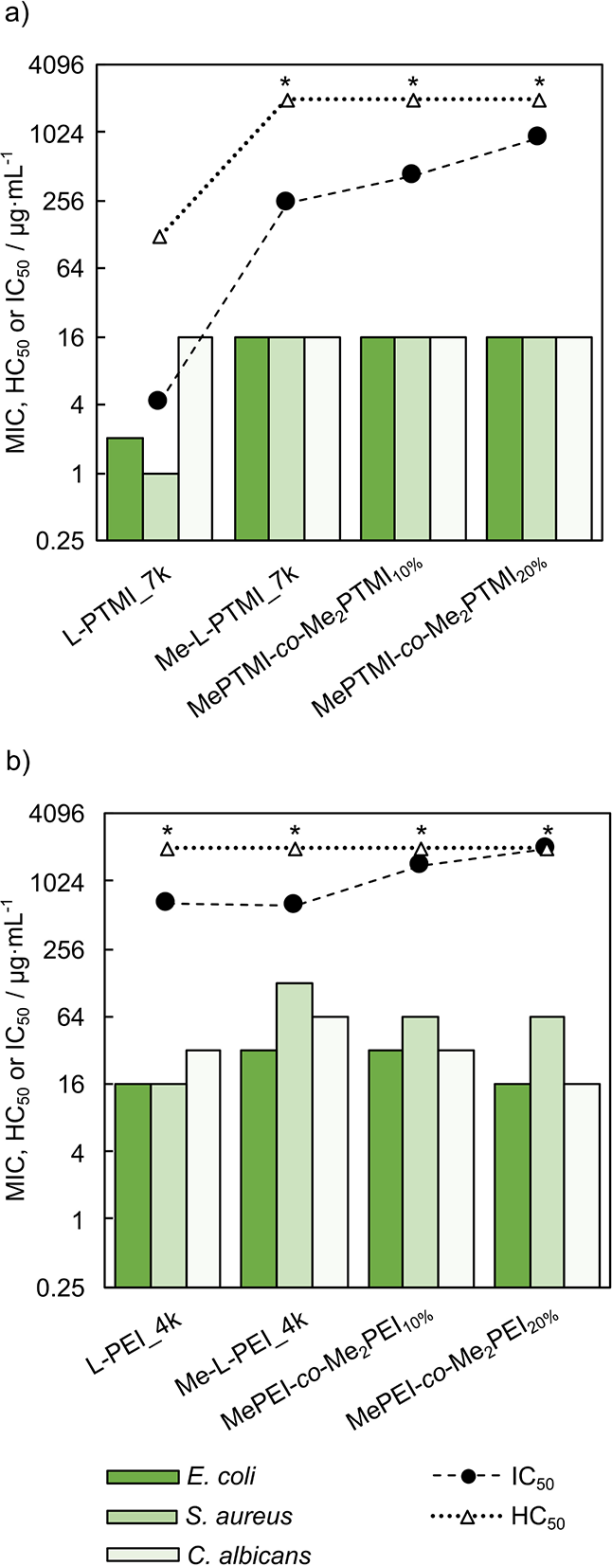
Values of MIC against model microorganisms, HC_50_, and IC_50_ (dashed line was drawn only to guide the eye) on different levels of postpolymerization modification for derivatives of (a) L-PTMI_7k and (b) L-PEI_4k (graphical representation); *HC_50_ > 2000 μg·mL^–1^.

The *N*-monomethylation of both L-PTMI and L-PEI leads to decreased antibacterial activity (Table 6, Figure 4). This effect is stronger for *S. aureus* than for *E. coli* and weakest for *C. albicans*. For example, the MIC values for Me-L-PTMI_7k against *E. coli* and *S. aureus* rise 8 and 16 times, respectively, compared to L-PTMI. The MIC value for Me-L-PEI_4k against *S. aureus* increases 8 times compared to unmodified L-PEI_4k, whereas the MIC against *E. coli* increases 2-fold (from 16 to 32 μg·mL^–1^). The *N*-monomethylation does not change the toxicity of L-PEI but significantly reduces the cytotoxicity and hemolytic activity of L-PTMI.

It is likely that *N*-monomethylation limits hydrogen bond formation and accessibility of the lone electron pair. The steric hindrance may be the reason for weakened interactions between the polymer and phospholipids in the cell membrane. However, other factors, such as an increased hydrophobicity of the molecule or a lack of direct chemical reactivity of tertiary amines (methylated polymers) toward carboxylic esters (amidation), could also play a role.^56^

Partial *N,N*-dimethylation of Me-L-PTMI_7k and Me-L-PEI_4k was performed to introduce a permanent cationic charge on the amine groups. Secondary and tertiary amines contain a cationic charge that depends on the level of protonation and is strictly linked to the pH of an environment. Quaternization provides a permanent charge and can improve electrostatic attraction between the polymer and the bacterial surface.

The partial *N,N*-dimethylation of L-PTMI_7k (DQ = 10% and DQ = 20%) does not change antimicrobial activity in comparison to *N*-methylated L-PTMI_7k (Figure 4a). The antimicrobial activity of Me-L-PEI_4k tends to slightly increase with the level of *N,N*-dimethylation for all tested microorganisms (Figure 4b), and the increase is the most pronounced for *C. albicans*. Quaternization of the studied polymers was expected to lead to a higher affinity to the cell surface, stronger binding to its anionic components, and improved antimicrobial activity. However, we do not observe this effect for L-PTMI, which may be due to the DQ being too low to induce a noticeable change in activity or the initial level of polymer protonation being high enough to mask any additional charge contribution. The postmodified L-PTMI_7k and L-PEI_4k do not show hemolytic activity at tested concentrations (2 mg·mL^–1^) (Table 6), which may be due to the increased hydrophilicity of the polymers.^96^

Interestingly, although methylation and partial quaternization do not improve antimicrobial properties of L-PTMI derivatives, they strongly reduce the cytotoxicity of these polymers (Figure 4a). As a result, some of them display substantial selectivity (Table 6, Figure S17). For postmodified L-PEI, quaternization also results in lower cytotoxicity (Figure 4b). This effect, combined with the increase in antimicrobial activity, results in a selectivity index for MePEI-*co*-Me_2_PEI_20%_ exceeding 100 in case of *E. coli* and *C. albicans* (Table 6, Figure S17).

### Activity against Clinical Isolates

Three polymers (namely, L-PTMI_1.2 k, L-PTMI_7k, and MePTMI-*co*-Me_2_PTMI_20%_) were selected to assay MIC values against clinical isolates of species belonging to the ESKAPE group^97^ that pose a particular medical threat by evading commonly used antibiotics. All polymers display substantial activity against clinical isolates with MIC values as low as 4 μg·mL^–1^, and it seems that investigated molecules present higher activity against Gram-positive than Gram-negative bacteria (Table 8). L-PTMI_7k displays lower MIC values than L-PTMI_1.2 k and partially quaternized L-PTMI_7k. These results are highly consistent with our observations for model microorganisms, where higher MWs and nonmodified polymers are more active. Importantly, L-PTMI_1.2 k and L-PTMI_7k retain good activity against clinically isolated *S. aureus*, both methicillin-sensitive or methicillin-resistant species. Similarly, no difference in MIC values can be observed between various strains of *K. pneumoniae* that are sensitive and resistant to β-lactam antibiotics. The same activity against β-lactam resistant and susceptible strains also supports the hypothesis that the mechanism of activity of the investigated polymers is linked to the disruption of the bacterial membrane.

**Table 8:** Antimicrobial Activity against Clinical Isolates

	MIC/μg·mL^–1^		
species	L-PTMI_7k	L-PTMI_1.2k	MePTMI-*co*-Me_2_PTMI_20%_
*E. coli* (2533/22)	8	128	32
*P. aeruginosa* (5037/22)	4	32	32
*A. baumannii* (MDR,^a^ 3109/19)	16	64	128
*K. pneumoniae* (5223/21)	16	32	128
*K. pneumoniae* (ESBL,^b^ 7128/21)	16	64	256
*K. pneumoniae* (ESBL,^b^ + CL-R,^c^ 6024/21)	16	64	512
*K. pneumoniae* (NDM,^d^ 3402/21)	16	64	64
*E. faecium* (VRE,^e^ 6877/20)	16	16	128
*S. aureus* (MRSA,^f^ 2218/21)	4	8	32
*S. aureus* (MRSA,^f^ 3040/21)	4	8	32
*S. aureus* (MRSA,^f^ 2914/21)	4	8	16
*S. aureus* (MSSA,^g^ 4114/19)	16	8	32
*S. aureus* (MSSA,^g^ 4855/19)	4	8	8
*S. aureus* (MSSA,^g^ 4679/19)	4	8	16
*S. aureus* (MSSA,^g^ 4938/19)	4	16	32

a Multidrug-resistant.

b Extended-spectrum β-lactamases.

c Ciprofloxacin-resistant.

d New Delhi metallo-β-lactamase.

e Vancomycin-resistant *Enterococcus*.

f Methicillin-resistant *Staphylococcus aureus*.

g Methicillin-sensitive *Staphylococcus aureus*.

Additionally, the polymers obtained in this work were tested against five different strains of *Mycobacterium tuberculosis* (Table 9), including isoniazid (INH) and rifampicin (RMP) resistant.^98^ The investigated polymers are substantially less active against *Mycobacterium* than against studied Gram-negative and Gram-positive strains. Some limited activity can be observed for polymers with low molecular weights. The large difference in activity between *Mycobacteria* and other bacteria could be explained by significant differences in the cell wall structure. It is likely that the *Mycobacterium* cell wall, which is rich in mycolic acids and arabinogalactan, limits penetration of the investigated polymers into the cell membrane surface and prevents interactions with cell membrane components.

**Table 9:** Values of MIC against *Mycobacterium tuberculosis*

	MIC/μg·mL^–1^				
polymer	H37Rv	INH^a^ resistant (7153)	RMP^b^ resistant (262/19)	INH and RMP resistant (561/17)	5419
L-PTMI_0.8k	>512	256	>512	>512	256
L-PTMI_1.2k	>512	256	>512	>512	>512
L-PTMI_3k	>512	512	>512	64	>512
L-PTMI_7k	>512	512	>512	>512	>512
L-PTMI_12k	>512	>512	>512	>512	>512
L-PTMI_18k	>512	>512	>512	>512	>512
Me-L-PTMI_7k	>512	>512	>512	>512	>512
MePTMI-*co*-Me_2_PTMI_20%_	>512	>512	>512	>512	>512
Me-L-PEI_4k	>512	>512	512	>512	>512
MePEI-*co*-Me_2_PEI_20%_	>512	>512	>512	>512	>512
INH	0.06	0.5	0.12	32	0.07
RMP	0.25	0.25	256	128	0.12

a Isoniazid.

b Rifampicin.

## Conclusions

In this work, we investigated a series of polymeric antimicrobial agents, namely, polyamines L-PEI and L-PTMI, and their derivatives. L-PTMI appears to be an interesting novel agent, active against Gram-positive and Gram-negative bacteria but with a moderate activity against *C. albicans* and no activity against *M. tuberculosis*.

For the first time, the impact of L-PTMI molecular weight on its antimicrobial activity has been demonstrated and compared with literature data for L-PEI. The polymer activity increases along with an increase of the polymer MW within the tested range (0.8–18 kDa). Importantly, MIC values of L-PTMI are as low as 1 μg·mL^–1^ against *S. aureus* and 4 μg·mL^–1^ against clinically isolated *P. aeruginosa* and methicillin-resistant *S. aureus*.

For both antimicrobial activity (MIC) and toxicity (IC_50_ and HC_50_) of L-PTMI, crossing a certain polymer weight substantially increases the activity of the macromolecule. Importantly, this MW threshold is located at lower masses for MIC values compared to HC_50_ and IC_50_. This provides an opportunity to utilize MW as a potential source of selectivity for novel polymeric antimicrobial agents.

L-PTMI is significantly more active than its analogue, L-PEI, when polymers with a similar number of r.u. are compared. Increasing the distance between amines in the polymer main chain from two to three methylene groups is beneficial for the antimicrobial activity of studied materials. Further postpolymerization modification of the polymers, namely, *N*-monomethylation and subsequent partial quaternization, typically reduces their antibacterial activity but also toxicity. As a result, those molecules bear substantial selectivity.

The materials obtained could serve as a starting point for active ingredients in disinfectants, antimicrobial films, or topical medical agents. More invasive uses such as wound dressing or intravenous applications would require further optimization of the structure to reduce toxicity.

## Supporting Information Available

^1^H NMR spectra, kinetic plots of polymerizations, SEC trace examples, additional graphical representations of MIC and selectivity for studied series of polymers, Hill equation, and uncertainty of IC_50_ (PDF)

## Footnotes

# In the literature, this polymer is called polytrimethylenimine (PTMI), polypropylenimine (PPI), or polyazetidine; in this work, we decided to use PTMI.

## References

[ref1] O’Neill J. Antimicrobial Resistance: Tackling a Crisis for the Health and Wealth of Nations The Review on Antimicrobial Resistance; 2014.

[ref2] Xie S. X.; Song L.; Yuca E.; Boone K.; Sarikaya R.; Vanoosten S. K.; Misra A.; Ye Q.; Spencer P.; Tamerler C. Antimicrobial Peptide-Polymer Conjugates for Dentistry. ACS Appl. Polym. Mater. 2020, 2, 1134–1144. 10.1021/acsapm.9b00921. 33834166 PMC8026165

[ref3] Palermo E. F.; Sovadinova I.; Kuroda K. Structural Determinants of Antimicrobial Activity and Biocompatibility in Membrane-Disrupting Methacrylamide Random Copolymers. Biomacromolecules 2009, 10, 3098–3107. 10.1021/bm900784x. 19803480

[ref4] Zhou C.; Yuan Y.; Zhou P.; Wang F.; Hong Y.; Wang N.; Xu S.; Du J. Highly Effective Antibacterial Vesicles Based on Peptide-Mimetic Alternating Copolymers for Bone Repair. Biomacromolecules 2017, 18, 4154–4162. 10.1021/acs.biomac.7b01209. 29020450

[ref5] Boschert D.; Schneider-Chaabane A.; Himmelsbach A.; Eickenscheidt A.; Lienkamp K. Synthesis and Bioactivity of Polymer-Based Synthetic Mimics of Antimicrobial Peptides (SMAMPs) Made from Asymmetrically Disubstituted Itaconates. Chem. – Eur. J. 2018, 24, 8217–8227. 10.1002/chem.201800907. 29600579 PMC7611503

[ref6] Salas-Ambrosio P.; Tronnet A.; Verhaeghe P.; Bonduelle C. Synthetic Polypeptide Polymers as Simplified Analogues of Antimicrobial Peptides. Biomacromolecules 2021, 22, 57–75. 10.1021/acs.biomac.0c00797. 32786537

[ref7] Liu S.; Ono R. J.; Wu H.; Teo J. Y.; Liang Z. C.; Xu K.; Zhang M.; Zhong G.; Tan J. P. K.; Ng M.; Yang C.; Chan J.; Ji Z.; Bao C.; Kumar K.; Gao S.; Lee A.; Fevre M.; Dong H.; Ying J. Y.; Li L.; Fan W.; Hedrick J. L.; Yang Y. Y. Highly Potent Antimicrobial Polyionenes with Rapid Killing Kinetics, Skin Biocompatibility and in Vivo Bactericidal Activity. Biomaterials 2017, 127, 36–48. 10.1016/j.biomaterials.2017.02.027. 28279920

[ref8] Yuan Y.; Liang S.; Li J.; Zhang S.; Zhang Y. Copolymers with Both Soft and Rigid Cationic Rings as Highly Selective Antimicrobials to Combat Antibiotic Resistant Microbes and Biofilms. J. Mater. Chem. B 2019, 7, 5620–5625. 10.1039/C9TB01264H. 31508648

[ref9] Lienkamp K.; Madkour A. E.; Tew G. N. Antibacterial Peptidomimetics: Polymeric Synthetic Mimics of Antimicrobial Peptides. Adv. Polym. Sci. 2010, 251, 141–172. 10.1007/12_2010_85.

[ref10] Pearce A. K.; O’Reilly R. K. Polymers for Biomedical Applications: The Importance of Hydrophobicity in Directing Biological Interactions and Application Efficacy. Biomacromolecules 2021, 22, 4459–4469. 10.1021/acs.biomac.1c00434. 34495643

[ref11] Gibney K. A.; Sovadinova I.; Lopez A. I.; Urban M.; Ridgway Z.; Caputo G. A.; Kuroda K. Poly(Ethylene Imine)s as Antimicrobial Agents with Selective Activity. Macromol. Biosci. 2012, 12, 1279–1289. 10.1002/mabi.201200052. 22865776 PMC3970578

[ref12] Lan T.; Guo Q.; Shen X. Polyethyleneimine and Quaternized Ammonium Polyethyleneimine: The Versatile Materials for Combating Bacteria and Biofilms. J. Biomater. Sci., Polym. Ed. 2019, 30, 1243–1259. 10.1080/09205063.2019.1627650. 31177926

[ref13] Yuan W.; Li H. Polymer-Based Nanocarriers for Therapeutic Nucleic Acids Delivery. In Nanostructures for Drug Delivery; Andronescu E.; Grumezescu A. M., Eds.; Elsevier, 2017; pp. 445–460.

[ref14] Kwon D. H.; Lu C. D. Polyamine Effects on Antibiotic Susceptibility in Bacteria. Antimicrob. Agents Chemother. 2007, 51, 2070–2077. 10.1128/AAC.01472-06. 17438056 PMC1891406

[ref15] Yao X.; Lu C. D. Characterization of Staphylococcus Aureus Responses to Spermine Stress. Curr. Microbiol. 2014, 69, 394–403. 10.1007/s00284-014-0603-y. 24816537

[ref16] Cheng W.; Yang C.; Ding X.; Engler A. C.; Hedrick J. L.; Yang Y. Y. Broad-Spectrum Antimicrobial/Antifouling Soft Material Coatings Using Poly(Ethylenimine) as a Tailorable Scaffold. Biomacromolecules 2015, 16, 1967–1977. 10.1021/acs.biomac.5b00359. 26039032

[ref17] Mikula P.; Mlnarikova M.; Takahashi H.; Babica P.; Kuroda K.; Blaha L.; Sovadinova I. Branched Poly(Ethylene Imine)s as Anti-Algal and Anti-Cyanobacterial Agents with Selective Flocculation Behavior to Cyanobacteria over Algae. Macromol. Biosci. 2018, 18, 1800187 10.1002/mabi.201800187. 30156762

[ref18] D’Angelo I.; Perfetto B.; Costabile G.; Ambrosini V.; Caputo P.; Miro A.; D’Emmanuele Di Villa Bianca R.; Sorrentino R.; Donnarumma G.; Quaglia F.; Ungaro F. Large Porous Particles for Sustained Release of a Decoy Oligonucelotide and Poly(Ethylenimine): Potential for Combined Therapy of Chronic Pseudomonas Aeruginosa Lung Infections. Biomacromolecules 2016, 17, 1561–1571. 10.1021/acs.biomac.5b01646. 27002689

[ref19] Demirci G.; Guven M. N.; Altuncu S.; Konca Y. U.; Avci D.; Yagci Acar H. (Bis)Phosphonic Acid-Functionalized Poly(Ethyleneimine)-Poly(Amido Amine)s for Selective in Vitro Transfection of Osteosarcoma Cells. ACS Appl. Polym. Mater. 2021, 3, 3776–3787. 10.1021/acsapm.1c00297.

[ref20] Chien H. W.; Chiu T. H.; Lee Y. L. Rapid Biocidal Activity of N-Halamine-Functionalized Polydopamine and Polyethylene Imine Coatings. Langmuir 2021, 37, 8037–8044. 10.1021/acs.langmuir.1c01256. 34160231

[ref21] Haldar J.; An D.; de Cienfuegos L. Á.; Chen J.; Klibanov A. M. Polymeric Coatings That Inactivate Both Influenza Virus and Pathogenic Bacteria. Proc. Natl. Acad. Sci. U. S. A. 2006, 103, 17667–17671. 10.1073/pnas.0608803103. 17101983 PMC1693804

[ref22] Stasko N. A.; Johnson C. B.; Schoenfisch M. H.; Johnson T. A.; Holmuhamedov E. L. Cytotoxicity of Polypropylenimine Dendrimer Conjugates on Cultured Endothelial Cells. Biomacromolecules 2007, 8, 3853–3859. 10.1021/bm7008203. 18004811

[ref23] Noske S.; Karimov M.; Aigner A.; Ewe A. Tyrosine-Modification of Polypropylenimine (PPI) and Polyethylenimine (PEI) Strongly Improves Efficacy of SiRNA-Mediated Gene Knockdown. Nanomaterials 2020, 10, 1809 10.3390/nano10091809. 32927826 PMC7557430

[ref24] Menzel H.; Horstmann S.; Behrens P.; Bärnreuther P.; Krueger I.; Jahns M. Chemical Properties of Polyamines with Relevance to the Biomineralization of Silica. Chem. Commun. 2003, 3, 2994–2995. 10.1039/B310201G. 14703824

[ref25] Suzuki T.; Shinkai S.; Sada K. Supramolecular Crosslinked Linear Poly (Trimethylene Iminium Trifluorosulfonimide) Polymer Gels Sensitive to Light and Thermal Stimuli. Adv. Mater. 2006, 18, 1043–1046. 10.1002/adma.200502552.

[ref26] Fischer W.; Brissault B.; Prévost S.; Kopaczynska M.; Andreou I.; Janosch A.; Gradzielski M.; Haag R. Synthesis of Linear Polyamines with Different Amine Spacings and Their Ability to Form DsDNA/SiRNA Complexes Suitable for Transfection. Macromol. Biosci. 2010, 10, 1073–1083. 10.1002/mabi.201000082. 20715130

[ref27] Bloksma M. M.; Schubert U. S.; Hoogenboom R. Poly(Cyclic Imino Ether)s beyond 2-Substituted-2-Oxazolines. Macromol. Rapid Commun. 2011, 32, 1419–1441. 10.1002/marc.201100138. 21714027

[ref28] Pang S. H.; Lively R. P.; Jones C. W. Oxidatively-Stable Linear Poly(Propylenimine)-Containing Adsorbents for CO2 Capture from Ultradilute Streams. ChemSusChem 2018, 11, 2628–2637. 10.1002/cssc.201800438. 29809307

[ref29] Hashimoto S.; Yamashita T. Synthesis of Linear Poly(1-Benzyltrimethyleneimine) by the Hofmann Reaction of Poly(1-Cyanoethyltrimethyleneimine) Quaternized with Benzylbromide. J. Macromol. Sci., Chem. 1991, 28, 475–486. 10.1080/00222339108052101.

[ref30] Yamashita T.; Furukawa I. Novel Syntheses of Poly(N-Alkyltrimethyleneimine)s by a Replacement of Cyanoethyl Groups in Poly(N-β-Cyanoethyltrimethyleneimine) with Other Alkyl Groups. J. Macromol. Sci., Part A: Pure Appl.Chem. 1997, 34, 843–853. 10.1080/10601329708014335.

[ref31] Elzes M. R.; Mertens I.; Sedlacek O.; Verbraeken B.; Doensen A. C. A.; Mees M. A.; Glassner M.; Jana S.; Paulusse J. M. J.; Hoogenboom R. Linear Poly(Ethylenimine-Propylenimine) Random Copolymers for Gene Delivery: From Polymer Synthesis to Efficient Transfection with High Serum Tolerance. Biomacromolecules 2022, 23, 2459–2470. 10.1021/acs.biomac.2c00210. 35499242

[ref32] Lambermont-Thijs H. M. L.; Heuts J. P. A.; Hoeppener S.; Hoogenboom R.; Schubert U. S. Selective Partial Hydrolysis of Amphiphilic Copoly(2-Oxazoline)s as Basis for Temperature and PH Responsive Micelles. Polym. Chem. 2011, 2, 313–322. 10.1039/C0PY00052C.

[ref33] Tanaka R.; Ueoka I.; Takaki Y.; Kataoka K.; Saito S. High Molecular Weight Linear Poly(Ethylenimine) and Poly(N-Methylethylenimine). Macromolecules 1983, 16, 849–853. 10.1021/ma00240a003.

[ref34] Zahoranová A.; Luxenhofer R. Poly(2-Oxazoline)- and Poly(2-Oxazine)-Based Self-Assemblies, Polyplexes, and Drug Nanoformulations—An Update. Adv. Healthcare Mater. 2021, 10, 2001382 10.1002/adhm.202001382. PMC11468752 33448122

[ref35] Park J.-R.; Verderosa A. D.; Totsika M.; Hoogenboom R.; Dargaville T. R. Thermoresponsive Polymer–Antibiotic Conjugates Based on Gradient Copolymers of 2-Oxazoline and 2-Oxazine. Biomacromolecules 2021, 22, 5185–5194. 10.1021/acs.biomac.1c01133. 34726387

[ref36] Hoogenboom R. The Future of Poly(2-Oxazoline)s. Eur. Polym. J. 2022, 179, 111521 10.1016/j.eurpolymj.2022.111521.

[ref37] Morgese G.; Verbraeken B.; Ramakrishna S. N.; Gombert Y.; Cavalli E.; Rosenboom J. G.; Zenobi-Wong M.; Spencer N. D.; Hoogenboom R.; Benetti E. M. Chemical Design of Non-Ionic Polymer Brushes as Biointerfaces: Poly(2-Oxazine)s Outperform Both Poly(2-Oxazoline)s and PEG. Angew. Chem., Int. Ed. 2018, 57, 11667–11672. 10.1002/anie.201805620. 30047615

[ref38] Varanaraja Z.; Kim J.; Becer C. R. Poly(2-Oxazine)s: A Comprehensive Overview of the Polymer Structures, Physical Properties and Applications. Eur. Polym. J. 2021, 147, 110299 10.1016/j.eurpolymj.2021.110299.

[ref39] Yang H.; Lopina S. T. Penicillin V-Conjugated PEG-PAMAM Star Polymers. J. Biomater. Sci., Polym. Ed. 2003, 14, 1043–1056. 10.1163/156856203769231556. 14661878

[ref40] Mauro N.; Schillaci D.; Varvarà P.; Cusimano M. G.; Geraci D. M.; Giuffrè M.; Cavallaro G.; Maida C. M.; Giammona G. Branched High Molecular Weight Glycopolypeptide with Broad-Spectrum Antimicrobial Activity for the Treatment of Biofilm Related Infections. ACS Appl. Mater. Interfaces 2018, 10, 318–331. 10.1021/acsami.7b16573. 29251486

[ref41] Romanovska A.; Keil J.; Tophoven J.; Oruc M. F.; Schmidt M.; Breisch M.; Sengstock C.; Weidlich D.; Klostermeier D.; Tiller J. C. Conjugates of Ciprofloxacin and Amphiphilic Block Copoly(2-Alkyl-2-Oxazolines)s Overcome Efflux Pumps and Are Active against CIP-Resistant Bacteria. Mol. Pharmaceutics 2021, 18, 3532–3543. 10.1021/acs.molpharmaceut.1c00430. 34323492

[ref42] Ganewatta M. S.; Tang C. Controlling Macromolecular Structures towards Effective Antimicrobial Polymers. Polymer 2015, 63, A1–A29. 10.1016/j.polymer.2015.03.007.

[ref43] Kanazawa A.; Ikeda T.; Endo T. Polymeric Phosphonium Salts as a Novel Class of Cationic Biocides. IV. Synthesis and Antibacterial Activity of Polymers with Phosphonium Salts in the Main Chain. J. Polym. Sci., Part A: Polym. Chem. 1993, 31, 3031–3038. 10.1002/pola.1993.080311219.

[ref44] Albert M.; Feiertag P.; Hayn G.; Saf R.; Hönig H. Structure - Activity Relationships of Oligoguanidines - Influence of Counterion, Diamine, and Average Molecular Weight on Biocidal Activities. Biomacromolecules 2003, 4, 1811–1817. 10.1021/bm0342180. 14606913

[ref45] Seyfarth F.; Schliemann S.; Elsner P.; Hipler U. C. Antifungal Effect of High- and Low-Molecular-Weight Chitosan Hydrochloride, Carboxymethyl Chitosan, Chitosan Oligosaccharide and N-Acetyl-d-Glucosamine against Candida Albicans, Candida Krusei and Candida Glabrata. Int. J. Pharm. 2008, 353, 139–148. 10.1016/j.ijpharm.2007.11.029. 18164151

[ref46] Zuo H.; Wu D.; Fu R. Synthesis of Antibacterial Polymers from 2-Dimethylamino Ethyl Methacrylate Quaternized by Dimethyl Sulfate. Polym. J. 2010, 42, 766–771. 10.1038/pj.2010.63.

[ref47] Kuroda K.; DeGrado W. F. Amphiphilic Polymethacrylate Derivatives as Antimicrobial Agents. J. Am. Chem. Soc. 2005, 127, 4128–4129. 10.1021/ja044205+. 15783168

[ref48] Lienkamp K.; Madkour A. E.; Kumar K.-N.; Nüsslein K.; Tew G. N. Antimicrobial Polymers Prepared by Ring-Opening Metathesis Polymerization: Manipulating Antimicrobial Properties by Organic Counterion and Charge Density Variation. Chem. – Eur. J. 2009, 15, 11715–11722. 10.1002/chem.200900606. 19798715

[ref49] Chen Y.; Wilbon P. A.; Chen Y. P.; Zhou J.; Nagarkatti M.; Wang C.; Chu F.; Decho A. W.; Tang C. Amphipathic Antibacterial Agents Using Cationic Methacrylic Polymers with Natural Rosin as Pendant Group. RSC Adv. 2012, 2, 10275 10.1039/c2ra21675b.

[ref50] Yang X.; Hu K.; Hu G.; Shi D.; Jiang Y.; Hui L.; Zhu R.; Xie Y.; Yang L. Long Hydrophilic-and-Cationic Polymers: A Different Pathway toward Preferential Activity against Bacterial over Mammalian Membranes. Biomacromolecules 2014, 15, 3267–3277. 10.1021/bm5006596. 25068991

[ref51] Panarin E. F.; Solovskii M. V.; Zaikina N. A.; Afinogenov G. E. Biological Activity of Cationic Polyelectrolytes. Makromol. Chem. 1985, 9, 25–33. 10.1002/macp.1985.020091985104.

[ref52] Mowery B. P.; Lindner A. H.; Weisblum B.; Stahl S. S.; Gellman S. H. Structure-Activity Relationships among Random Nylon-3 Copolymers That Mimic Antibacterial Host-Defense Peptides. J. Am. Chem. Soc. 2009, 131, 9735–9745. 10.1021/ja901613g. 19601684

[ref53] Kuroda K.; Caputo G. A.; DeGrado W. F. The Role of Hydrophobicity in the Antimicrobial and Hemolytic Activities of Polymethacrylate Derivatives. Chem. – Eur. J. 2009, 15, 1123–1133. 10.1002/chem.200801523. 19072946 PMC3814040

[ref54] Locock K. E. S.; Michl T. D.; Valentin J. D. P.; Vasilev K.; Hayball J. D.; Qu Y.; Traven A.; Griesser H. J.; Meagher L.; Haeussler M. Guanylated Polymethacrylates: A Class of Potent Antimicrobial Polymers with Low Hemolytic Activity. Biomacromolecules 2013, 14, 4021–4031. 10.1021/bm401128r. 24099527

[ref55] Liu R.; Chen X.; Falk S. P.; Mowery B. P.; Karlsson A. J.; Weisblum B.; Palecek S. P.; Masters K. S.; Gellman S. H. Structure-Activity Relationships among Antifungal Nylon-3 Polymers: Identification of Materials Active against Drug-Resistant Strains of Candida Albicans. J. Am. Chem. Soc. 2014, 136, 4333–4342. 10.1021/ja500036r. 24606327 PMC3985965

[ref56] Monnery B. D.; Wright M.; Cavill R.; Hoogenboom R.; Shaunak S.; Steinke J. H. G.; Thanou M. Cytotoxicity of Polycations: Relationship of Molecular Weight and the Hydrolytic Theory of the Mechanism of Toxicity. Int. J. Pharm. 2017, 521, 249–258. 10.1016/j.ijpharm.2017.02.048. 28232268

[ref57] Tyagi A.; Mishra A. Optimal Balance of Hydrophobic Content and Degree of Polymerization Results in a Potent Membrane-Targeting Antibacterial Polymer. ACS Omega 2021, 6, 34724–34735. 10.1021/acsomega.1c05148. 34963955 PMC8697380

[ref58] Mayandi V.; Sridhar S.; Fazil M. H. U. T.; Goh E. T. L.; Htoon H. M.; Orive G.; Choong Y. K.; Saravanan R.; Beuerman R. W.; Barkham T. M. S.; Yang L.; Baskaran M.; Jhanji V.; Loh X. J.; Verma N. K.; Lakshminarayanan R. Protective Action of Linear Polyethylenimine against Staphylococcus Aureus Colonization and Exaggerated Inflammation in Vitro and in Vivo. ACS Infect. Dis. 2019, 5, 1411–1422. 10.1021/acsinfecdis.9b00102. 31099239

[ref59] Witte H.; Seeliger W. Simple Synthesis of 2-Substituted 2-Oxazolines and 5,6-Dihydro-4H-1,3-oxazines. Angew. Chem., Int. Ed. Engl. 1972, 11, 287–288. 10.1002/anie.197202871.

[ref60] Bloksma M. M.; Paulus R. M.; van Kuringen H. P. C.; van der Woerdt F.; Lambermont-Thijs H. M. L.; Schubert U. S.; Hoogenboom R. Thermoresponsive Poly(2-Oxazine)s. Macromol. Rapid Commun. 2012, 33, 92–96. 10.1002/marc.201100587. 22121033

[ref61] Lambermont-Thijs H. M. L.; Fijten M. W. M.; Van Der Linden A. J.; Van Lankvelt B. M.; Bloksma M. M.; Schubert U. S.; Hoogenboom R. Efficient Cationic Ring-Opening Polymerization of Diverse Cyclic Imino Ethers: Unexpected Copolymerization Behavior. Macromolecules 2011, 44, 4320–4325. 10.1021/ma200426y.

[ref62] Sedlacek O.; Lava K.; Verbraeken B.; Kasmi S.; de Geest B. G.; Hoogenboom R. Unexpected Reactivity Switch in the Statistical Copolymerization of 2-Oxazolines and 2-Oxazines Enabling the One-Step Synthesis of Amphiphilic Gradient Copolymers. J. Am. Chem. Soc. 2019, 141, 9617–9622. 10.1021/jacs.9b02607. 31136165

[ref63] Monnery B. D.; Shaunak S.; Thanou M.; Steinke J. H. G. Improved Synthesis of Linear Poly(Ethylenimine) via Low-Temperature Polymerization of 2-Isopropyl-2-Oxazoline in Chlorobenzene. Macromolecules 2015, 48, 3197–3206. 10.1021/acs.macromol.5b00437.

[ref64] Lambermont-Thijs H. M. L.; van der Woerdt F. S.; Baumgaertel A.; Bonami L.; du Prez F. E.; Schubert U. S.; Hoogenboom R. Linear Poly(Ethylene Imine)s by Acidic Hydrolysis of Poly(2-Oxazoline)s: Kinetic Screening, Thermal Properties, and Temperature-Induced Solubility Transitions. Macromolecules 2010, 43, 927–933. 10.1021/ma9020455.

[ref65] Verbraeken B.; Monnery B. D.; Lava K.; Hoogenboom R. The Chemistry of Poly(2-Oxazoline)s. Eur. Polym. J. 2017, 88, 451–469. 10.1016/j.eurpolymj.2016.11.016.

[ref66] Seeliger W.; Aufderhaar E.; Diepers W.; Feinauer R.; Nehring R.; Thier W.; Hellmann H. Recent Syntheses and Reactions of Cyclic Imidic Esters. Angew. Chem., Int. Ed. Engl. 1966, 5, 875–888. 10.1002/anie.196608751. 4962631

[ref67] Hoogenboom R.; Fijten M. W. M.; Thijs H. M. L.; Van Lankvelt B. M.; Schubert U. S. Microwave-Assisted Synthesis and Properties of a Series of Poly(2-Alkyl-2-Oxazoline)s. Des. Monomers Polym. 2005, 8, 659–671. 10.1163/156855505774597704.

[ref68] Lambermont-Thijs H. M. L.; Bonami L.; Du Prez F. E.; Hoogenboom R. Linear Poly(Alkyl Ethylene Imine) with Varying Side Chain Length: Synthesis and Physical Properties. Polym. Chem. 2010, 1, 747–754. 10.1039/b9py00344d.

[ref69] Kopiasz R. J.; Szczepańczyk M.; Jańczewski D. Unusual Enhancement of Degradation Rate Induced by Polymer Chain Elongation in Quaternized Polyethyleneimine Derivatives. React. Funct. Polym. 2019, 137, 96–103. 10.1016/j.reactfunctpolym.2019.01.010.

[ref70] Kopiasz R. J.; Kozon D.; Pachla J.; Skórka Ł.; Jańczewski D. Controlled Post-Polymerization Modification through Modulation of Repeating Unit Reactivity: Proof of Concept Discussed Using Linear Polyethylenimine Example. Polymer 2021, 217, 123452 10.1016/j.polymer.2021.123452.

[ref71] Lienkamp K.; Madkour A. E.; Musante A.; Nelson C. F.; Nüsslein K.; Tew G. N. Antimicrobial Polymers Prepared by ROMP with Unprecedented Selectivity: A Molecular Construction Kit Approach. J. Am. Chem. Soc. 2008, 130, 9836–9843. 10.1021/ja801662y. 18593128 PMC4106262

[ref72] Xing H.; Lu M.; Yang T.; Liu H.; Sun Y.; Zhao X.; Xu H.; Yang L.; Ding P. Structure-Function Relationships of Nonviral Gene Vectors : Lessons from Antimicrobial Polymers. Acta Biomater. 2019, 86, 15–40. 10.1016/j.actbio.2018.12.041. 30590184

[ref73] Ikeda T.; Hirayama H.; Yamaguchi H.; Tazuke S.; Watanabe M. Polycationic Biocides with Pendant Active Groups: Molecular Weight Dependence of Antibacterial Activity. Antimicrob. Agents Chemother. 1986, 30, 132–136. 10.1128/AAC.30.1.132. 3092730 PMC176450

[ref74] Kenawy E. R.; Worley S. D.; Broughton R. The Chemistry and Applications of Antimicrobial Polymers: A State-of-the-Art Review. Biomacromolecules 2007, 8, 1359–1384. 10.1021/bm061150q. 17425365

[ref75] Rabea E. I.; Badawy M. E. T.; Stevens C. V.; Smagghe G.; Steurbaut W. Chitosan as Antimicrobial Agent: Applications and Mode of Action. Biomacromolecules 2003, 4, 1457–1465. 10.1021/bm034130m. 14606868

[ref76] Kong M.; Chen X. G.; Xing K.; Park H. J. Antimicrobial Properties of Chitosan and Mode of Action: A State of the Art Review. Int. J. Food Microbiol. 2010, 144, 51–63. 10.1016/j.ijfoodmicro.2010.09.012. 20951455

[ref77] Ikeda T.; Yamaguchi H.; Tazuke S. Molecular Weight Dependence of Antibacterial Activity in Cationic Disinfectants. J. Bioact. Compat. Polym. 1990, 5, 31–41. 10.1177/088391159000500104.

[ref78] Ikeda T.; Ledwith A.; Bamford C. H.; Hann R. A. Interaction of a Polymeric Biguanide Biocide with Phospholipid Membranes. Biochim. Biophys. Acta, Biomembr. 1984, 769, 57–66. 10.1016/0005-2736(84)90009-9. 6691980

[ref79] Wiegand C.; Bauer M.; Hipler U. C.; Fischer D. Poly(Ethyleneimines) in Dermal Applications: Biocompatibility and Antimicrobial Effects. Int. J. Pharm. 2013, 456, 165–174. 10.1016/j.ijpharm.2013.08.001. 23948135

[ref80] Ilker M. F.; Nüsslein K.; Tew G. N.; Coughlin E. B. Tuning the Hemolytic and Antibacterial Activities of Amphiphilic Polynorbornene Derivatives. J. Am. Chem. Soc. 2004, 126, 15870–15875. 10.1021/ja045664d. 15571411

[ref81] Shima S.; Matsuoka H.; Iwamoto T.; Sakai H. Antimicrobial Action of ε-Poly-L-Lysine. Jpn. J. Antibiot. 1984, 37, 1449–1455. 10.7164/antibiotics.37.1449. 6392269

[ref82] Fox S. J.; Fazil M. H. U. T.; Dhand C.; Venkatesh M.; Goh E. T. L.; Harini S.; Eugene C.; Lim R. R.; Ramakrishna S.; Chaurasia S. S.; Beuerman R. W.; Verma C. S.; Verma N. K.; Loh X. J.; Lakshminarayanan R. Insight into Membrane Selectivity of Linear and Branched Polyethylenimines and Their Potential as Biocides for Advanced Wound Dressings. Acta Biomater. 2016, 37, 155–164. 10.1016/j.actbio.2016.04.015. 27079762

[ref83] Kozon D.; Mierzejewska J.; Kobiela T.; Grochowska A.; Dudnyk K.; Głogowska A.; Sobiepanek A.; Kuźmińska A.; Ciach T.; Augustynowicz-Kopeć E.; Jańczewski D. Amphiphilic Polymethyloxazoline–Polyethyleneimine Copolymers: Interaction with Lipid Bilayer and Antibacterial Properties. Macromol. Biosci. 2019, 19, 1900254 10.1002/mabi.201900254. 31747130

[ref84] Venkatesh M.; Barathi V. A.; Goh E. T. L.; Anggara R.; Fazil M. H. U. T.; Ng A. J. Y.; Harini S.; Aung T. T.; Fox S. J.; Liu S.; Yang L.; Barkham T. M. S.; Loh X. J.; Verma N. K.; Beuerman R. W.; Lakshminarayanan R. Antimicrobial Activity and Cell Selectivity of Synthetic and Biosynthetic Cationic Polymers. Antimicrob. Agents Chemother. 2017, 61, e00469-17 10.1128/AAC.00469-17. 28784676 PMC5610535

[ref85] Richter L.; Hijazi M.; Arfeen F.; Krumm C.; Tiller J. C. Telechelic, Antimicrobial Hydrophilic Polycations with Two Modes of Action. Macromol. Biosci. 2018, 18, 1700389 10.1002/mabi.201700389. 29512268

[ref86] Falco A.; Encinas P.; Carbajosa S.; Cuesta A.; Chaves-Pozo E.; Tafalla C.; Estepa A.; Coll J. M. Transfection Improvements of Fish Cell Lines by Using Deacylated Polyethylenimine of Selected Molecular Weights. Fish Shellfish Immunol. 2009, 26, 559–566. 10.1016/j.fsi.2009.02.013. 19250970

[ref87] Gilbert P.; Moore L. E. Cationic Antiseptics: Diversity of Action under a Common Epithet. J. Appl. Microbiol. 2005, 99, 703–715. 10.1111/j.1365-2672.2005.02664.x. 16162221

[ref88] Grigor’eva M. N.; Stel’makh S. A.; Astakhova S. A.; Tsenter I. M.; Bazaron L. U.; Batoev V. B.; Mognonov D. M. Synthesis of Polyalkylguanidine Hydrochloride Copolymers and Their Antibacterial Activity Against Conditionally Pathogenic Microorganisms Bacillus Cereus and Escherichia Coli. Pharm. Chem. J. 2015, 49, 99–103. 10.1007/s11094-015-1230-z.

[ref89] Palermo E. F.; Lienkamp K.; Gillies E. R.; Ragogna P. J. Antibacterial Activity of Polymers: Discussions on the Nature of Amphiphilic Balance. Angew. Chem. 2019, 131, 3728–3731. 10.1002/ange.201813810. 30653795

[ref90] Tossi A.; Sandri L.; Giangaspero A. Amphipathic, α-Helical Antimicrobial Peptides. Biopolymers 2000, 55, 4–30. 10.1002/1097-0282(2000)55:1<4::AID-BIP30>3.0.CO;2-M. 10931439

[ref91] Walker L.; Sood P.; Lenardon M. D.; Milne G.; Olson J.; Jensen G.; Wolf J.; Casadevall A.; Adler-Moore J.; Gow N. A. R. The Viscoelastic Properties of the Fungal Cell Wall Allow Traffic of Ambisome as Intact Liposome Vesicles. MBio 2018, 9, e02383-17 10.1128/mBio.02383-17. 29437927 PMC5801470

[ref92] Halder S.; Yadav K. K.; Sarkar R.; Mukherjee S.; Saha P.; Haldar S.; Karmakar S.; Sen T. Alteration of Zeta Potential and Membrane Permeability in Bacteria: A Study with Cationic Agents. Springerplus 2015, 4, 672 10.1186/s40064-015-1476-7. 26558175 PMC4633473

[ref93] Chen H.; Zhou Y.; Zhou X.; Liao B.; Xu H. H. K.; Chu C. H.; Cheng L.; Ren B. Dimethylaminododecyl Methacrylate Inhibits Candida Albicans and Oropharyngeal Candidiasis in a PH-Dependent Manner. Appl. Microbiol. Biotechnol. 2020, 104, 3585–3595. 10.1007/s00253-020-10496-0. 32125481

[ref94] Ramamourthy G.; Park J.; Seo C.; Vogel H. J.; Park Y. Antifungal and Antibiofilm Activities and the Mechanism of Action of Repeating Lysine-Tryptophan Peptides against Candida Albicans. Microorganisms 2020, 8, 758 10.3390/microorganisms8050758. 32443520 PMC7285485

[ref95] Azevedo M. M.; Ramalho P.; Silva A. P.; Teixeira-Santos R.; Pina-Vaz C.; Rodrigues A. G. Polyethyleneimine and Polyethyleneimine-Based Nanoparticles: Novel Bacterial and Yeast Biofilm Inhibitors. J. Med. Microbiol. 2014, 63, 1167–1173. 10.1099/jmm.0.069609-0. 24913563

[ref96] Kopiasz R. J.; Rukasz A.; Chreptowicz K.; Podgórski R.; Kuźmińska A.; Mierzejewska J.; Tomaszewski W.; Ciach T.; Jańczewski D. Influence of Lipid Bilayer Composition on the Activity of Antimicrobial Quaternary Ammonium Ionenes, the Interplay of Intrinsic Lipid Curvature and Polymer Hydrophobicity, the Role of Cardiolipin. Colloids Surf., B 2021, 207, 112016 10.1016/j.colsurfb.2021.112016. 34364250

[ref97] Rice L. B. Federal Funding for the Study of Antimicrobial Resistance in Nosocomial Pathogens: No ESKAPE. J. Infect. Dis. 2008, 197, 1079–1081. 10.1086/533452. 18419525

[ref98] Phillips D. J.; Harrison J.; Richards S. J.; Mitchell D. E.; Tichauer E.; Hubbard A. T. M.; Guy C.; Hands-Portman I.; Fullam E.; Gibson M. I. Evaluation of the Antimicrobial Activity of Cationic Polymers against Mycobacteria: Toward Antitubercular Macromolecules. Biomacromolecules 2017, 18, 1592–1599. 10.1021/acs.biomac.7b00210. 28365981 PMC5435458

