# Supplementary material for: Polytrimethylenimines: Highly Potent Antibacterial Agents with Activity and Toxicity Modulated by the Polymer Molecular Weight
Source: Biomacromolecules. 2023 Apr 24;24(5):2237–49. doi: 10.1021/acs.biomac.3c00139 (PMC10170506; doi:10.1021/acs.biomac.3c00139)
Supplement: Supplementary file 1 — bm3c00139_si_001.pdf [file bm3c00139_si_001.pdf]

Supporting Information

Polytrimethylenimines: highly potent antibacterial agents with activity and toxicity modulated by the polymer molecular weight

Julita Pachla,[a] *Rafał J. Kopiasz,*[a] *Gabriela Marek,*[a] *Waldemar Tomaszewski,*[a] *Agnieszka Głogowska,*[c] *Karolina Dreżek,*[a] *Sebastian Kowalczyk,*[a] *Rafał Podgórski,*[b] *Beata Butruk-Raszeja,*[b] *Tomasz Ciach,*[b] *Jolanta Mierzejewska,*[a] *Andrzej Plichta,*[a] *Ewa Augustynowicz-Kopeć,*[c] *Dominik Jańczewski*[a] *

[a] Faculty of Chemistry, Warsaw University of Technology, Noakowskiego 3, 00-664 Warsaw, Poland

[b] Faculty of Chemical and Process Engineering, Warsaw University of Technology, Waryńskiego 1, 00-645 Warsaw, Poland

[c] Department of Microbiology, National Tuberculosis and Lung Diseases Research Institute, Płocka 26, Warsaw 01-138, Poland

Content

^{1}H NMR spectra 2
Kinetic Investigation of the Microwave-Assisted Polymerization 8
Size Exclusion Chromatography..9
Degree of polymerization of L-PTMI...10
Antimicrobial activity 10
Cytotoxicity...12
Selectivity 14
References...15

^{1}H NMR spectra

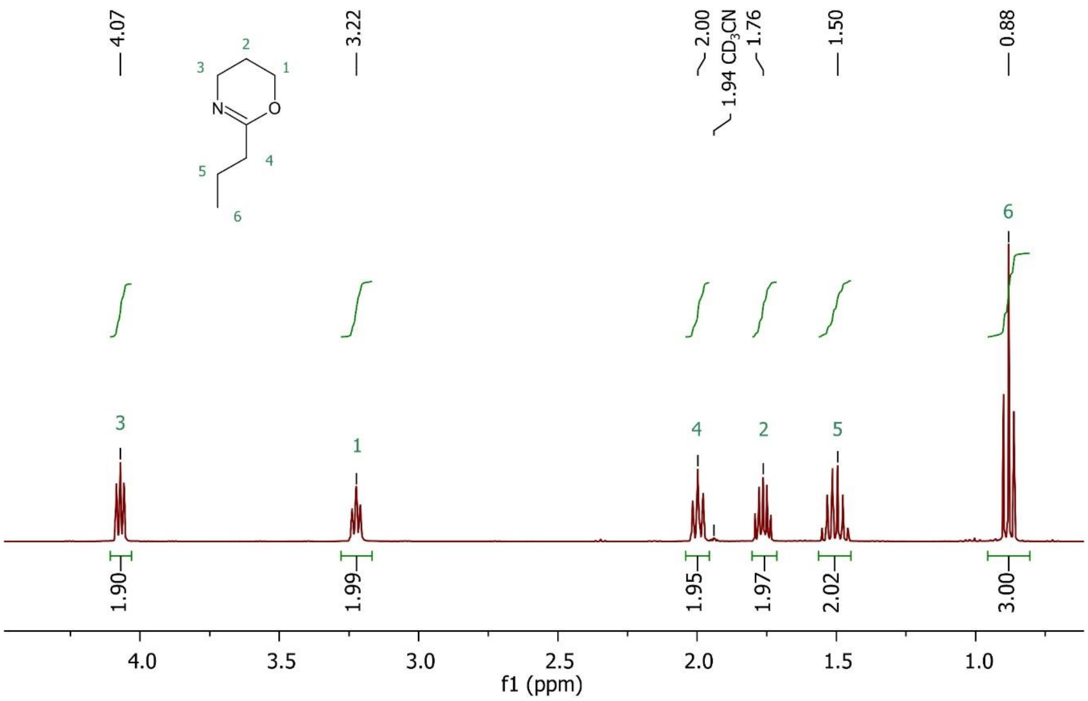

Figure S1. ^{1}H NMR spectrum of 2-*n*-propyl-2-oxazine (400 MHz, CD_3CN).

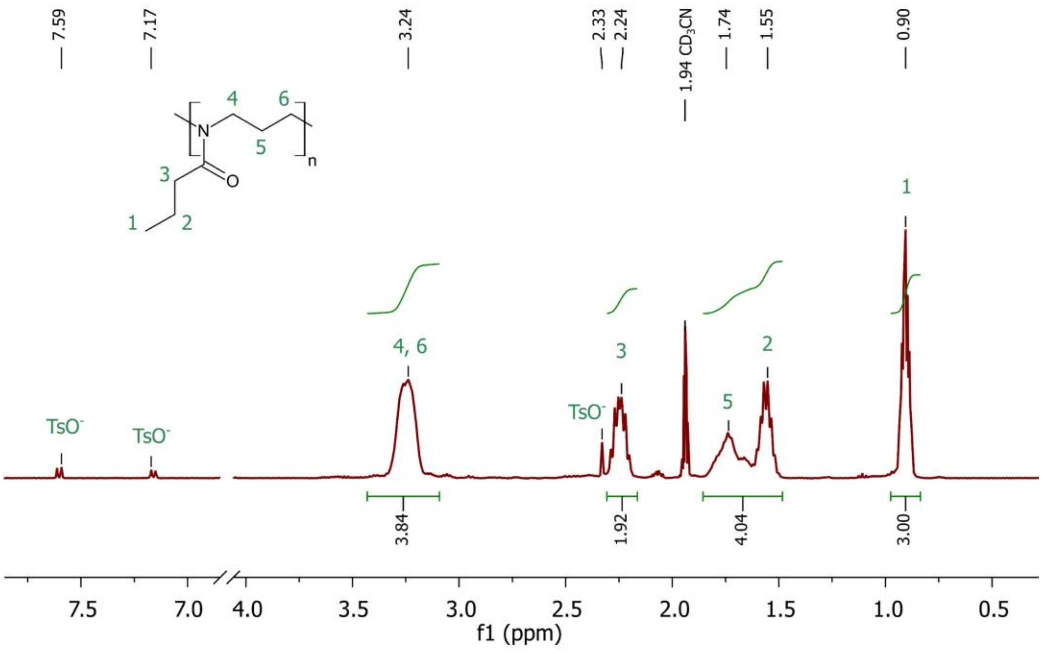

Figure S2. ^{1}H NMR spectrum PPrOzi_3k (400 MHz, CD_3CN).

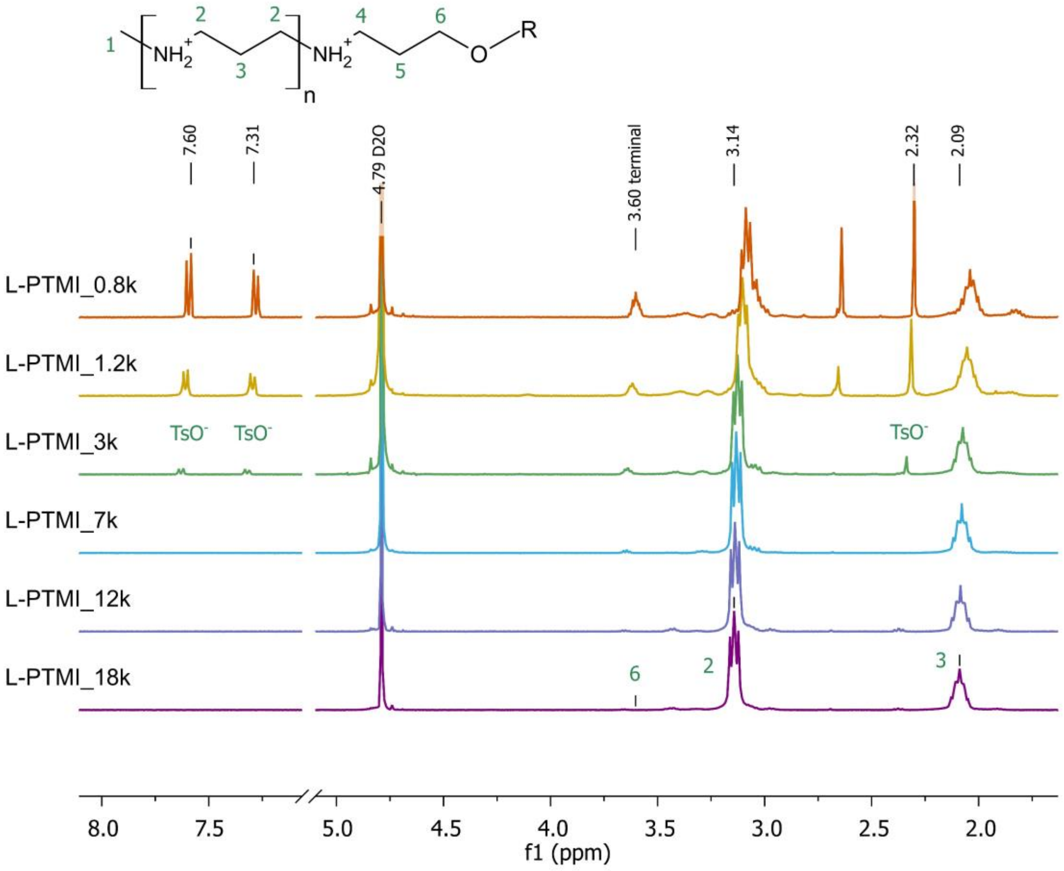

Figure S3. ^{1}H NMR spectra of L-PTMI series with different molecular weight (400 MHz, D_2O).

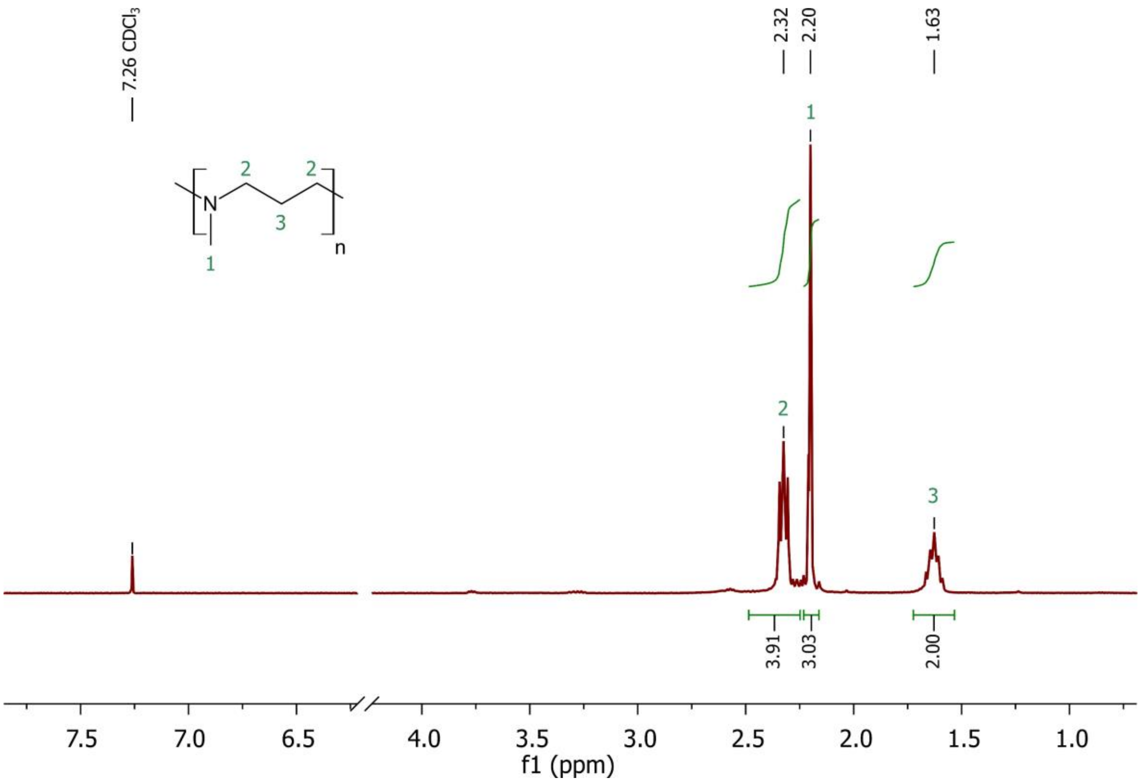

Figure S4. ^{1}H NMR spectrum of Me-L-PTMI_7k (400 MHz, $CDCl_3$).

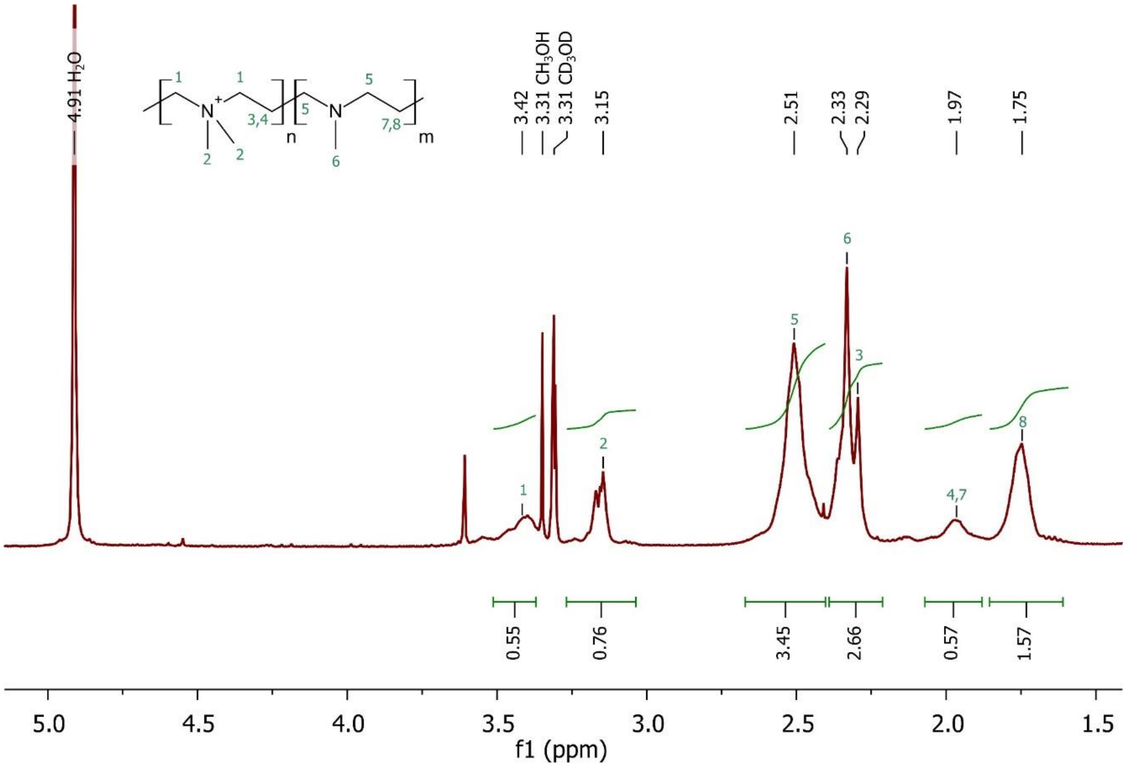

Figure S5. ^{1}H NMR spectrum of MePTMI-*co*-$Me_2PTMI_{10\%}$ (400 MHz, CD_3OD).

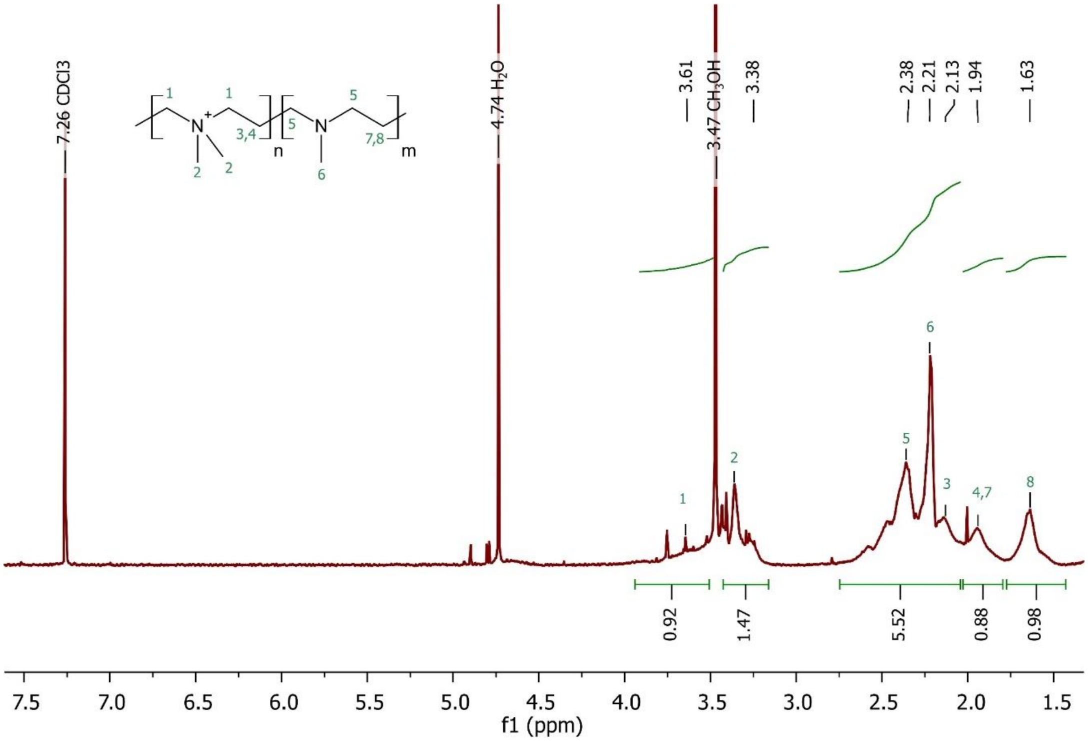

Figure S6. ^{1}H NMR spectrum of MePTMI-*co*-$Me_2$$PTMI_{20\%}$ (400 MHz, $CDCl_3$).

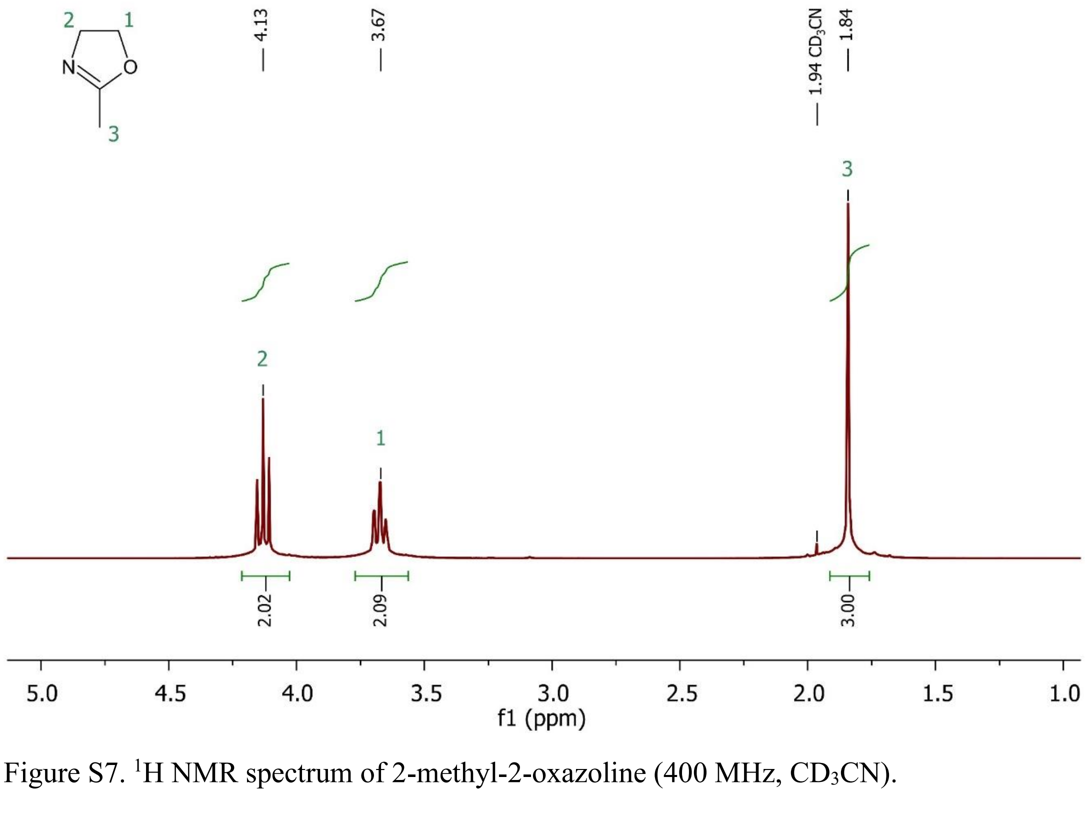

Figure S7. ^{1}H NMR spectrum of 2-methyl-2-oxazoline (400 MHz, CD_3CN).

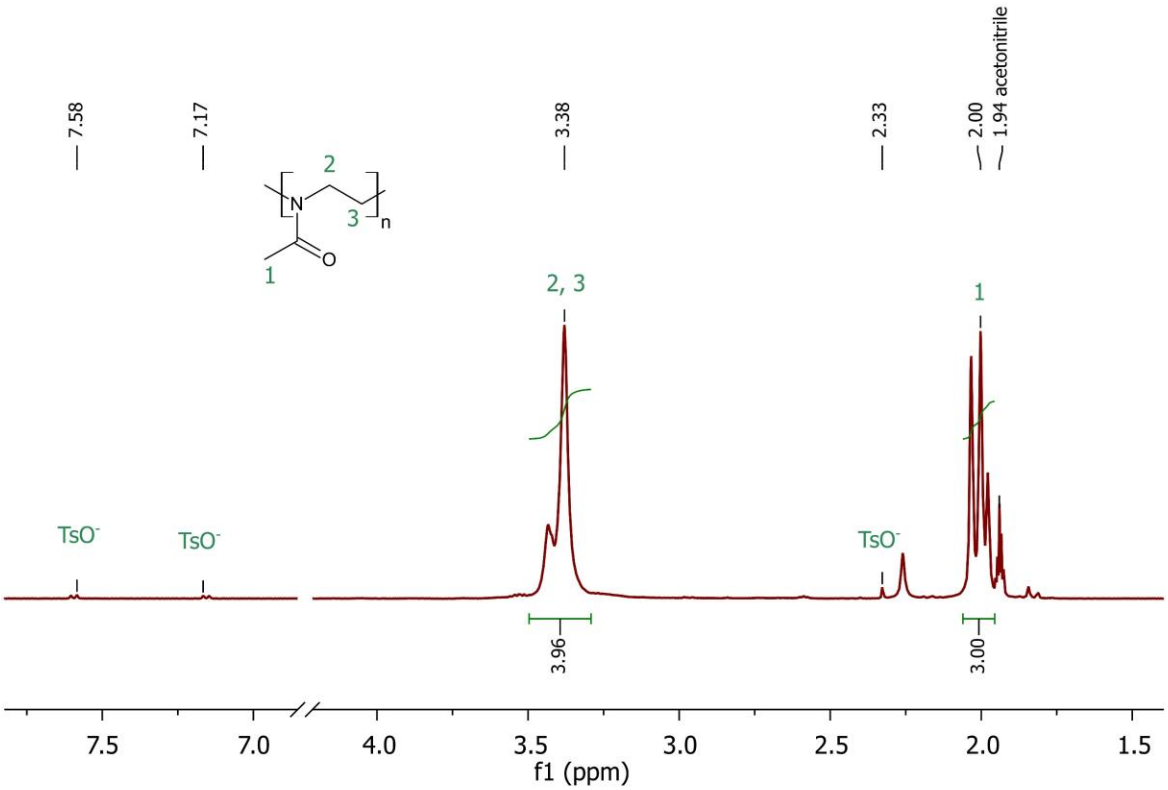

Figure S8. ^{1}H NMR spectrum of Poly(2-methyl-2-oxazoline) (PMeOx_4k) (400 MHz, CD_3CN).

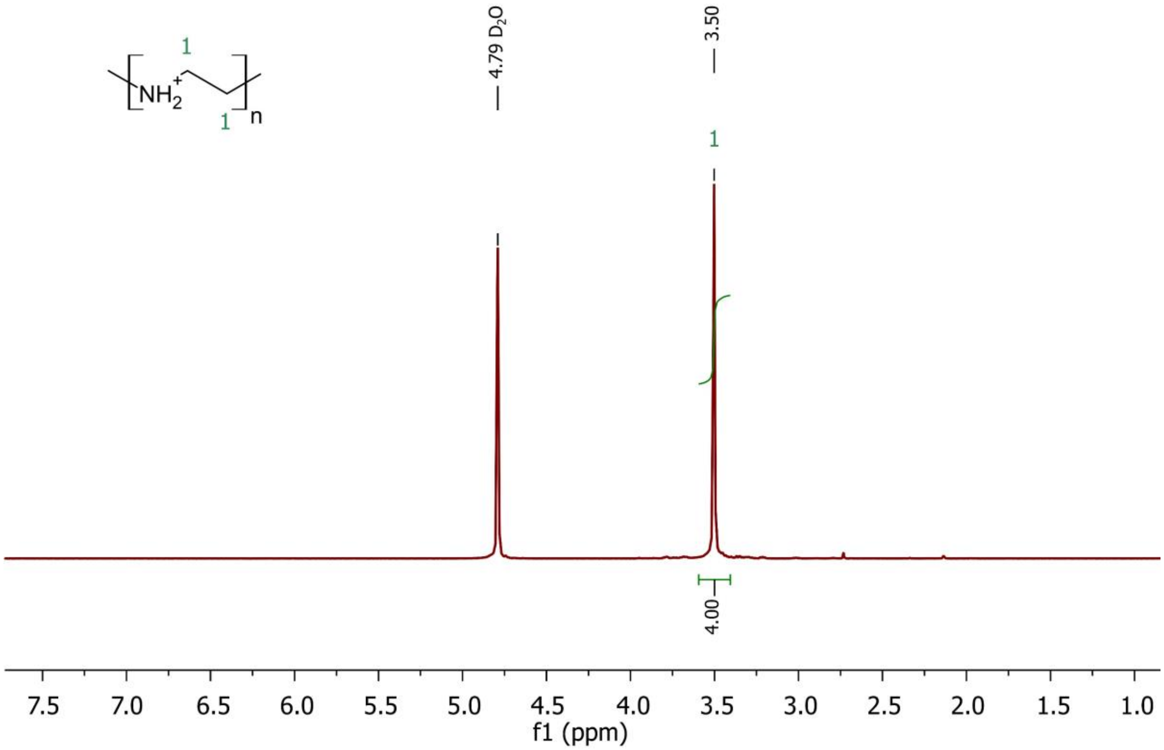

Figure S9. ^{1}H NMR spectrum of L-PEI_4k (400 MHz, D_2O).

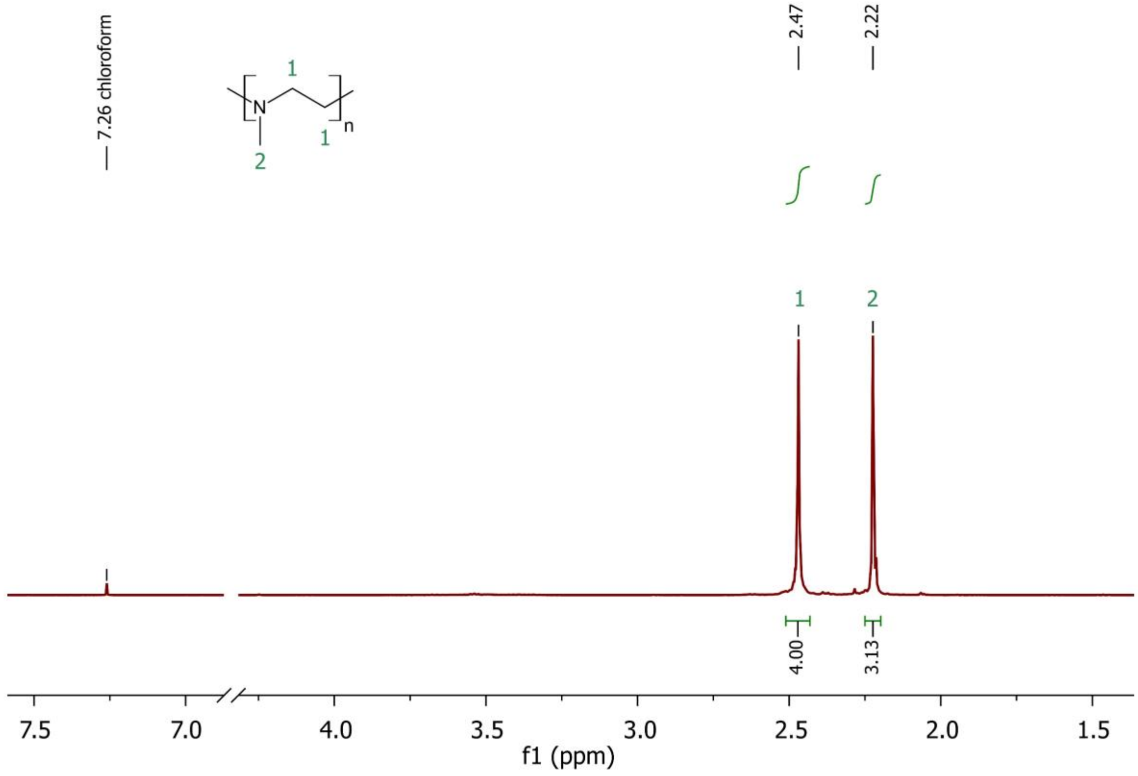

Figure S10. ^{1}H NMR spectrum of Me-L-PEI_4k (400 MHz, $CDCl_3$).

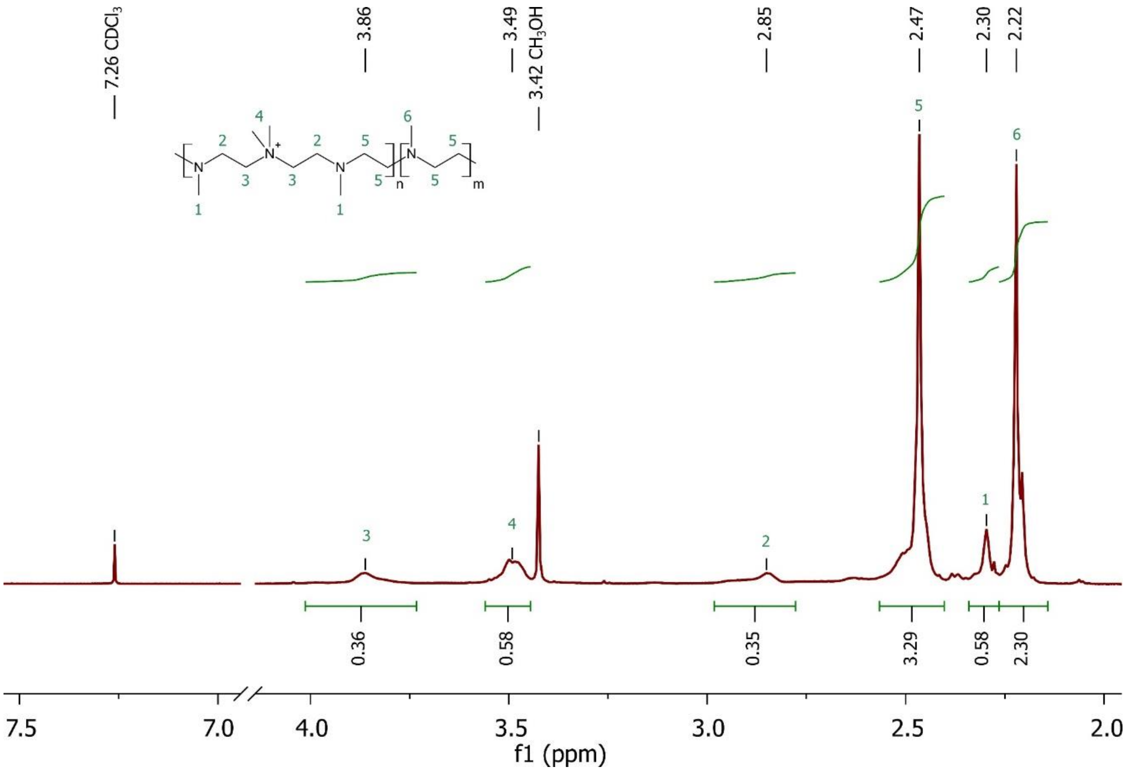

Figure S11. ^{1}H NMR spectrum of MePEI-co-$Me_2PEI_{10\%}$ (400 MHz, $CDCl_3$).

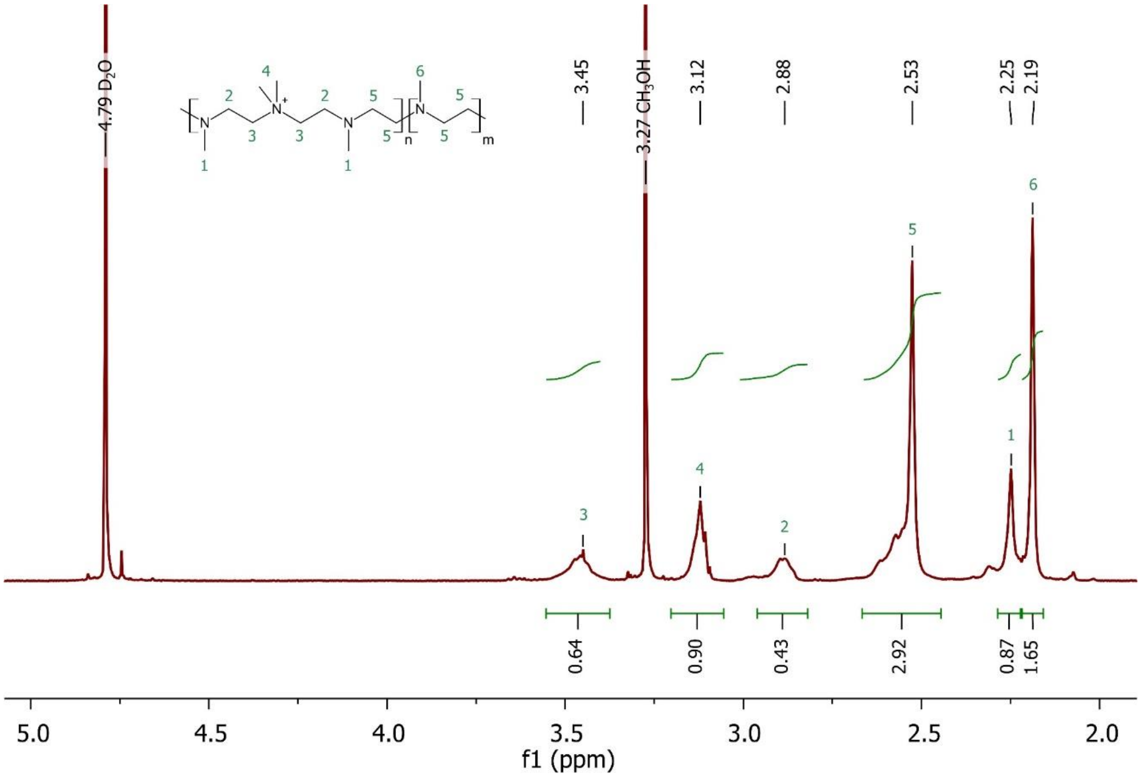

Figure S12. ^{1}H NMR spectrum of MePEI-co-$Me_2PEI_{20\%}$ (400 MHz, D_2O).

Kinetic Investigation of the Microwave-Assisted Polymerization

Kinetic investigations of polymerizations were performed under microwave irradiation at 120 °C with an initial monomer concentration of 4 M in acetonitrile and a monomer (2-*n*-propyl-2-oxazine or 2-methyl-2-oxazoline) to initiator (MeOTs) ratio of 100. A stock solution was prepared and divided over separate microwave reaction vessels (3 mL each) that were heated to 120 °C for different times. The conversion of monomers was measured by means of GC, to estimate the polymerization rates. Acetonitrile (HPLC grade, Sigma-Aldrich) was treated as an internal standard, as in each polymerization series constant ratio of reagents were used and ACN, as the solvent, was not consumed during reactions. Dimethylformamide (HPLC grade, POCh), was used as an eluent for GC without purification. The results are presented in Figure S13Figure S13.

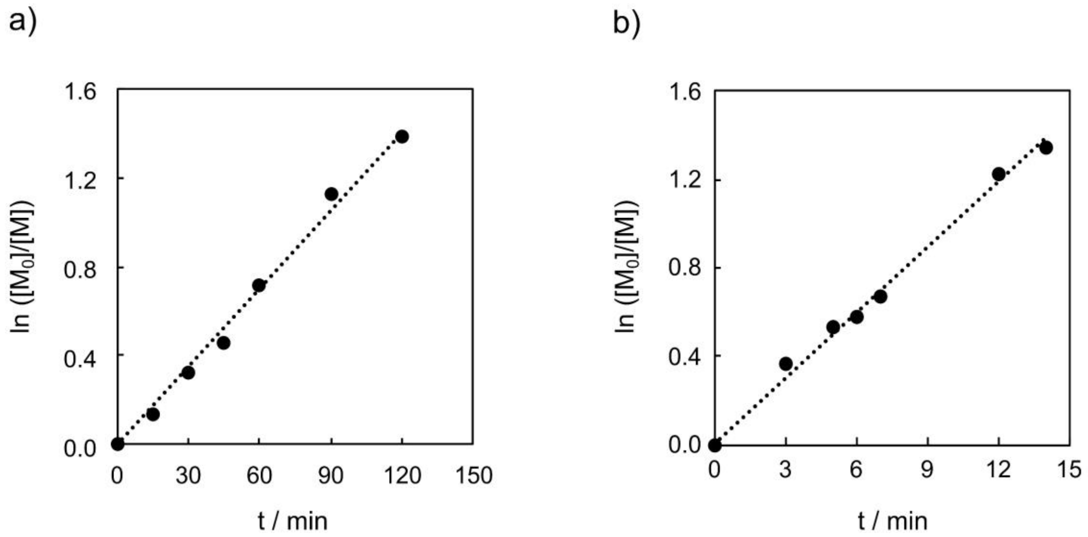

Figure S13. Kinetic plots for polymerizations initiated with MeOTs ($[M_0]/[I_0]$ = 100) in acetonitrile at 120 °C, a) 2-*n*-propyl-2-oxazine polymerizations, b) 2-methyl-2-oxazoline polymerizations.

Size Exclusion Chromatography

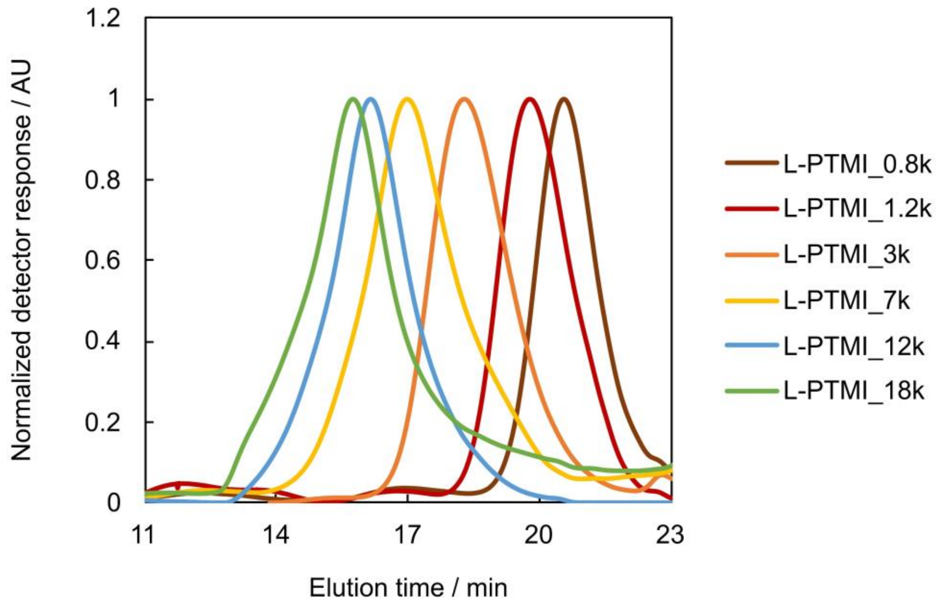

Figure S14. SEC traces of studied polymers from L-PTMI series.

Degree of polymerization of L-PTMI

DP_{NMR} was calculated as the ratio of integrals of signals in ^{1}H NMR spectra coming from the repeating unit (2.09 ppm, $-NH_2^+CH_2C\mathit{H_2}CH_2-$) and the terminal group (3.60 ppm, $-NH_2^+CH_2CH_2C\mathit{H_2}OR$). $DP_{predicted}$ was calculated based on the amount of used initiator, conversion of the monomer monitored by GC and assuming living character of the polymerization.

Table S1. Degrees of polymerization based on ^{1}H NMR (DP_{NMR}) and GC ($DP_{predicted}$).

Polymer	$M_{n,SEC}$ / kDa	DP_{NMR}	$DP_{predicted}$
L-PTMI_0.8k	0.80	4	5
L-PTMI_1.2k	1.2	7	13
L-PTMI_3k	2.8	12	32
L-PTMI_7k	6.7	48	51
L-PTMI_12k	12.1	96	67
L-PTMI_18k	18.0	144	75

Antimicrobial activity

Table S2. Literature and measured data, MIC values for oligomeric polyamines.

Compound	MIC / µg·mL^{-1}					
	E. coli		*S. aureus*		*C. albicans*	
	Literature data	This work	Literature data	This work	Literature data	This work
Spermine	202 – 404[1]	1024	808[1]	256	n.d. [a]	128
Spermidine	>2324,[1] >500,[2] >145[3]	2048	>2324,[1] >500[2]	512	>145[3]	512
Norspermidine	>131[3]	2048	n.d. [a]	512	>131[3]	512
Putrescine	250[2]	2048	250,[2] >4400[4]	1024	n.d. [a]	1024

[a] No data available.

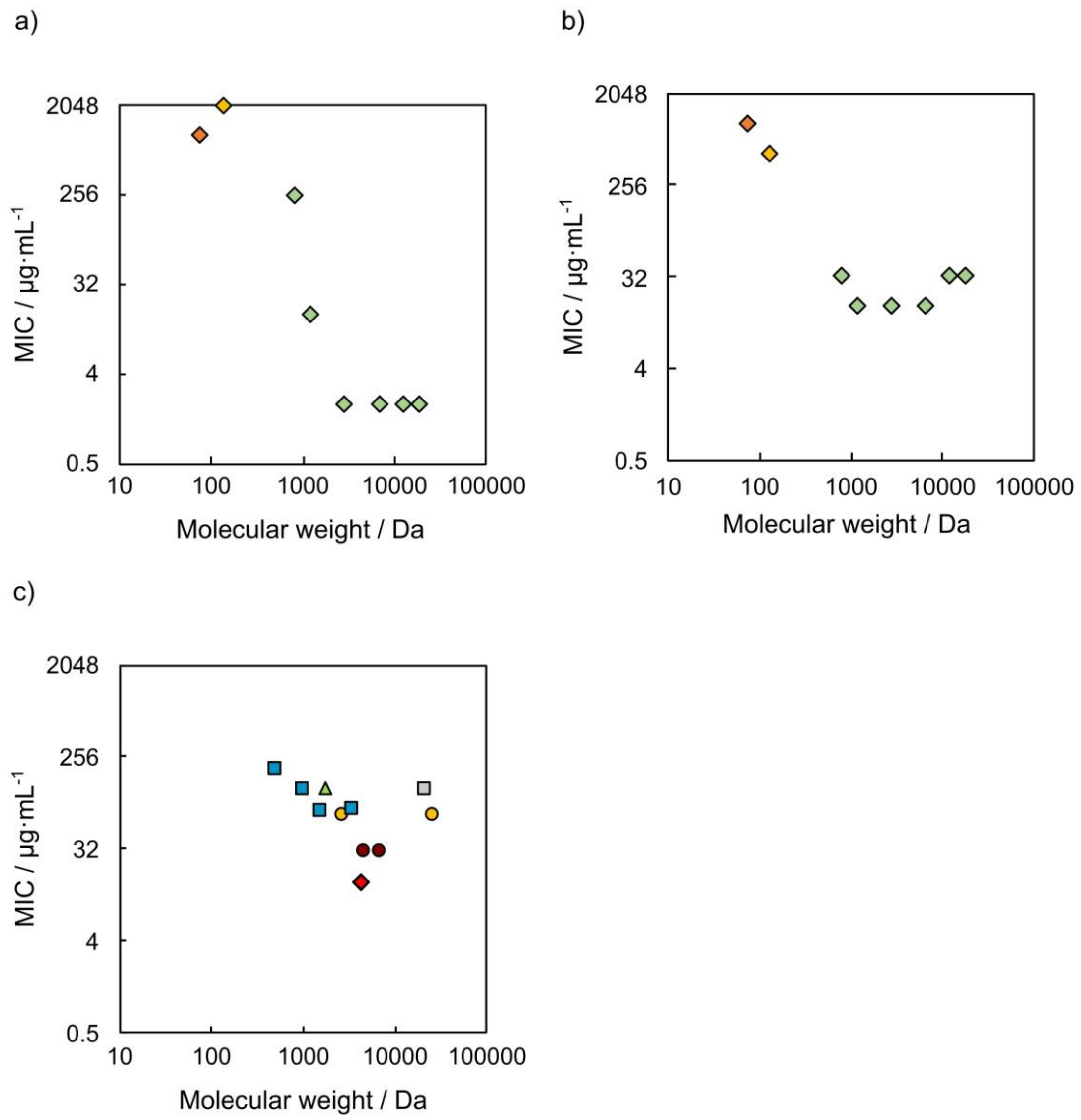

Figure S15. Minimum inhibitory concentration in function of molecular weight for linear polyamines a) L-PTMI and its oligomeric analogues with three methylene spacer against *E. coli*; b) L-PTMI and its oligomeric analogues with three methylene spacer against *C. albicans*; c) L-PEI (majority literature data; the legend: ◆ This work (L-PTMI); ◆ This work (norspermidine); ◆ This work (diaminopropane); ◆ This work (L-PEI); ● Gibney et al.;[5] ● Wiegand et al.;[6] ■ Fox et al.;[7] ■ Richter et al.;[8] ▲ Kozon et al.[9]

Cytotoxicity

The IC_{50} values were determined with the Quest Graph™ IC50 Calculator at https://www.aatbio.com/tools/ic50-calculator by fitting experimental data to the Hill equation using a four parameter logistic regression model:

$$\mathrm{A} = Min + \frac{Max - Min}{1 + \left(\frac{\mathrm{C}}{\mathrm{IC_{50}}}\right)^{\mathrm{n}}}$$

where:

A – absorbance at 475 nm under polymer concentration C

C – polymer concentration

IC_{50} – half maximal inhibitory concentration

n – Hill coefficient

Min – minimum value of absorbance

Max – maximum value of absorbance.

Absorbance was measured for each polymer for 8 different concentrations and mean values of absorbance were calculated from 6 repeats. Exemplary experimental points and model plot are presented in Figure S16. To determine the uncertainty of IC_{50} values, the standard deviation of log(IC_{50}) was calculated (Table S3).

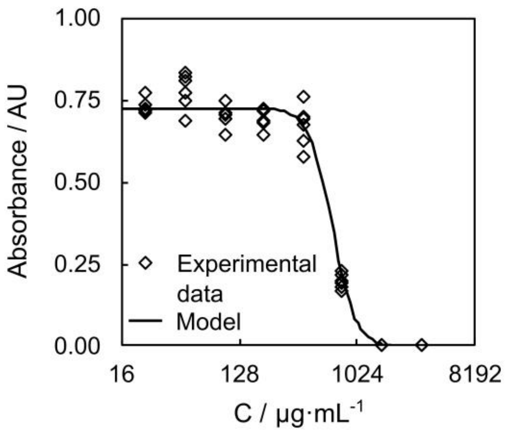

Figure S16. Representative dependence of absorbance on polymer concentration (L-PEI_4k).

Table S3. Uncertainties of cytotoxicity data.

Polymer	log IC_{50} ± SD[a]	IC_{50} ± SD[a] / $\mu g \cdot mL^{-1}$
L-PTMI_0.8k	3.346 ± 0.057	2220^{+310}_{-280}
L-PTMI_1.2k	3.411 ± 0.059	2570^{+380}_{-330}
L-PTMI_3k	1.402 ± 0.062	$25.2^{+3.9}_{-3.4}$
L-PTMI_7k	0.623 ± 0.048	$4.20^{+0.49}_{-0.44}$
L-PTMI_12k	0.395 ± 0.055	$2.48^{+0.33}_{-0.30}$
L-PTMI_18k	0.433 ± 0.065	$2.71^{+0.44}_{-0.38}$
L-PEI_4k	2.818 ± 0.056	660^{+91}_{-80}
Me-L-PTMI_7k	2.380 ± 0.080	240^{+49}_{-41}
MePTMI-*co*-$Me_2PTMI_{10\%}$	2.622 ± 0.060	420^{+62}_{-54}
MePTMI-*co*-$Me_2PTMI_{20\%}$	2.971 ± 0.073	930^{+170}_{-150}
Me-L-PEI_4k	2.78 ± 0.15	600^{+240}_{-170}
MePEI-*co*-$Me_2PEI_{10\%}$	3.138 ± 0.064	1370^{+220}_{-190}
MePEI-*co*-$Me_2PEI_{20\%}$	3.300 ± 0.059	2000^{+290}_{-260}

[a] Standard deviation.

Selectivity

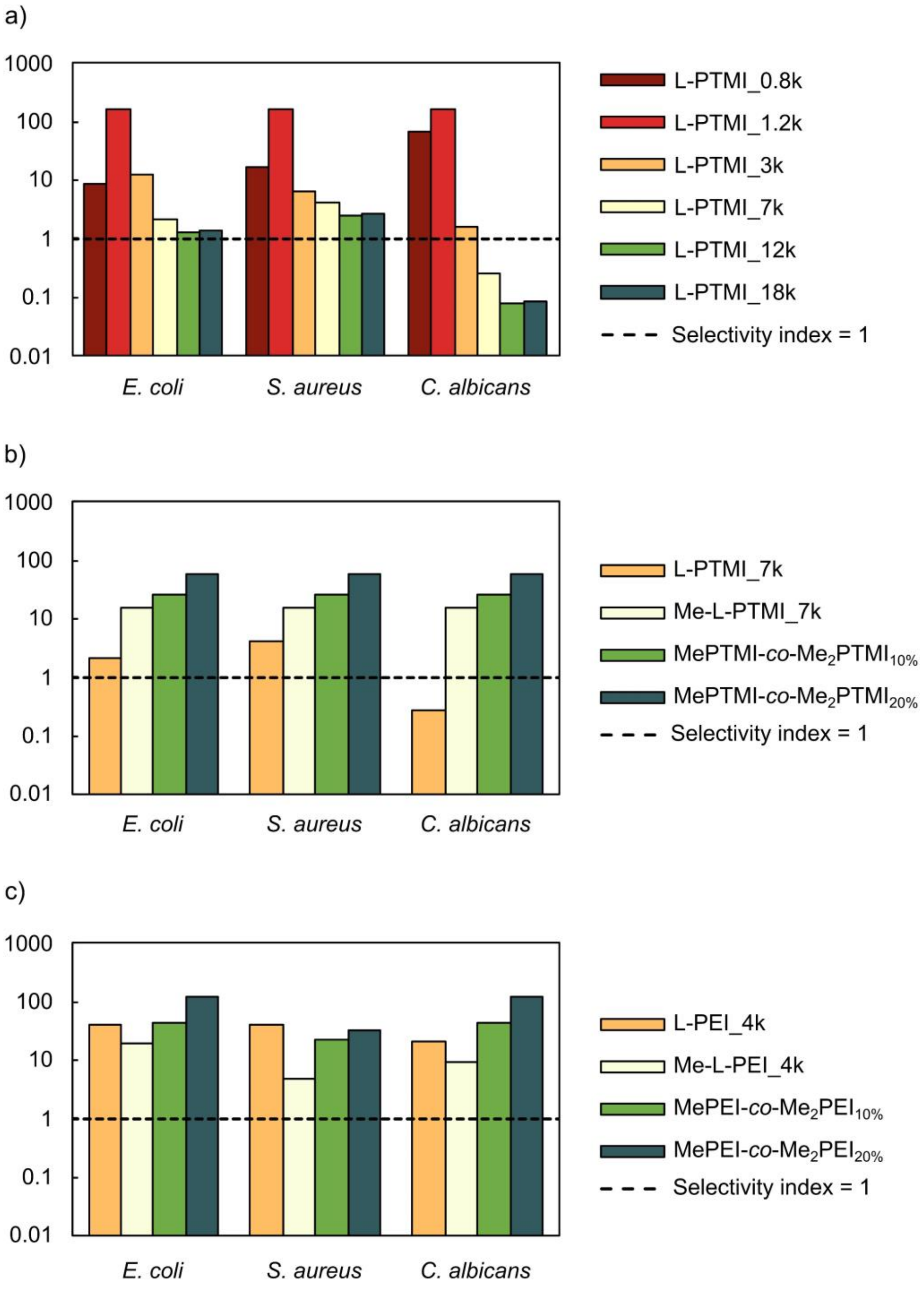

Figure S17. Selectivity against model microorganisms a) for L-PTMI with different molecular weight; b) for derivatives of L-PTMI_7k on the different level of post-polymerization modification; c) microorganisms for derivatives of L-PEI_4k on the different level of post-polymerization modification.

References

(1) Kwon, D. H.; Lu, C. D. Polyamine Effects on Antibiotic Susceptibility in Bacteria. *Antimicrob. Agents Chemother.* **2007**, *51* (6), 2070–2077.

(2) Gagic, M.; Kociova, S.; Smerkova, K.; Michalkova, H.; Setka, M.; Svec, P.; Pribyl, J.; Masilko, J.; Balkova, R.; Heger, Z.; Richtera, L.; Adam, V.; Milosavljevic, V. One-Pot Synthesis of Natural Amine-Modified Biocompatible Carbon Quantum Dots with Antibacterial Activity. *J. Colloid Interface Sci.* **2020**, *580*, 30–48.

(3) Takahashi, E.; Oono, S.; Yamamoto, S.; Arimoto, S.; Negishi, T.; Okamoto, K. Spermidine-Analogous Triamines Suppressed the Growth of Candida Albicans. *Biol. Pharm. Bull.* **1440**, *36* (9), 1440–1447.

(4) Razin, S.; Rozansky, R. Mechanism of the Antibacterial Action of Spermine. *Arch. Biochem. Biophys.* **1959**, *81*, 36–54.

(5) Gibney, K. A.; Sovadinova, I.; Lopez, A. I.; Urban, M.; Ridgway, Z.; Caputo, G. A.; Kuroda, K. Poly(Ethylene Imine)s as Antimicrobial Agents with Selective Activity. *Macromol. Biosci.* **2012**, *12* (9), 1279–1289.

(6) Wiegand, C.; Bauer, M.; Hipler, U. C.; Fischer, D. Poly(Ethyleneimines) in Dermal Applications: Biocompatibility and Antimicrobial Effects. *Int. J. Pharm.* **2013**, *456* (1), 165–174.

(7) Fox, S. J.; Fazil, M. H. U. T.; Dhand, C.; Venkatesh, M.; Goh, E. T. L.; Harini, S.; Eugene, C.; Lim, R. R.; Ramakrishna, S.; Chaurasia, S. S.; Beuerman, R. W.; Verma, C. S.; Verma, N. K.; Loh, X. J.; Lakshminarayanan, R. Insight into Membrane Selectivity of Linear and Branched Polyethylenimines and Their Potential as Biocides for Advanced Wound Dressings. *Acta Biomat.* **2016**, *37*, 155–164.

(8) Richter, L.; Hijazi, M.; Arfeen, F.; Krumm, C.; Tiller, J. C. Telechelic, Antimicrobial Hydrophilic Polycations with Two Modes of Action. *Macromol. Biosci.* **2018**, *18* (4), 1–9.

(9) Kozon, D.; Mierzejewska, J.; Kobiela, T.; Grochowska, A.; Dudnyk, K.; Głogowska, A.; Sobiepanek, A.; Kuźmińska, A.; Ciach, T.; Augustynowicz-Kopeć, E.; Jańczewski, D. Amphiphilic Polymethyloxazoline–Polyethyleneimine Copolymers: Interaction with Lipid Bilayer and Antibacterial Properties. *Macromol. Biosci.* **2019**, *19* (12), 1900254.
